# Characterization of QTL and Environmental Interactions Controlling Flowering Time in Andean Common Bean (*Phaseolus vulgaris* L.)

**DOI:** 10.3389/fpls.2020.599462

**Published:** 2021-01-14

**Authors:** Ana M. González, Fernando J. Yuste-Lisbona, Jim Weller, Jacqueline K. Vander Schoor, Rafael Lozano, Marta Santalla

**Affiliations:** ^1^Grupo de Genética del Desarrollo de Plantas, Misión Biológica de Galicia-CSIC, Pontevedra, Spain; ^2^Departamento de Biología y Geología (Genética), Centro de Investigación en Biotecnología Agroalimentaria (BITAL), Universidad de Almería, Almería, Spain; ^3^School of Natural Sciences, University of Tasmania, Hobart, TAS, Australia

**Keywords:** common bean, environment interaction, epistasis, flowering time, photoperiod, QTL

## Abstract

Genetic variation for response of flowering time to photoperiod plays an important role in adaptation to environments with different photoperiods, and as consequence is an important contributor to plant productivity and yield. To elucidate the genetic control of flowering time [days to flowering (DTF); growing degree days (GDD)] in common bean, a facultative short-day plant, a quantitative trait loci (QTL) analysis was performed in a recombinant inbred mapping population derived from a cultivated accession and a photoperiod sensitive landrace, grown in different long-day (LD) and short-day (SD) environments by using a multiple-environment QTL model approach. A total of 37 QTL across 17 chromosome regions and 36 QTL-by-QTL interactions were identified for six traits associated with time to flowering and response to photoperiod. The DTF QTL accounted for 28 and 11% on average of the phenotypic variation in the population across LD and SD environments, respectively. Of these, a genomic region on chromosome 4 harboring the major DTF QTL was associated with both flowering time in LD and photoperiod response traits, controlling more than 60% of phenotypic variance, whereas a major QTL on chromosome 9 explained up to 32% of flowering time phenotypic variation in SD. Different epistatic interactions were found in LD and SD environments, and the presence of significant QTL × environment (QE) and epistasis × environment interactions implies that flowering time control may rely on different genes and genetic pathways under inductive and non-inductive conditions. Here, we report the identification of a novel major locus controlling photoperiod sensitivity on chromosome 4, which might interact with other loci for controlling common bean flowering time and photoperiod response. Our results have also demonstrated the importance of these interactions for flowering time control in common bean, and point to the likely complexity of flowering time pathways. This knowledge will help to identify and develop opportunities for adaptation and breeding of this legume crop.

## Introduction

Flowering time control involves the regulation of physiological processes that are integrated and coordinated in a complex network with other developmental processes (Weller and Ortega, 2015). As a short-day plant (SDP), common bean (*Phaseolus vulgaris* L.) exhibits delayed flowering when grown in latitudes with longer summer daylengths (Garner and Allard, 1920). In addition, photoperiod is known to affect other vegetative and reproductive traits in common bean, such as stem elongation, branching, leaf morphology, floral architecture, and pod filling (Wallace, 1985). Historical selective pressures favoring improved production in non-favorable daylengths are manifested today as a major genetic differentiation between wild and domesticated common bean, and dramatically reduced photoperiod sensitivity in a proportion of accessions in each of the two major domesticated genepools (White and Laing, 1989). Like wild *P. vulgaris*, most Andean cultivars are photoperiod sensitive, while Mesoamerican and determinate cultivars include a high proportion of day-neutral lines (White and Laing, 1989). Temperature is another environmental factor influencing flowering time control in common bean, and is known to interact with photoperiod sensitivity, which increases at higher temperatures. At low latitudes, where the temperatures are relatively stable, responses to photoperiod are strongly responsive to temperature and largely reflect regional differences in altitude (Wallace, 1985; White and Laing, 1989). In contrast, germplasm adapted to higher latitudes, where day-to-day variation in temperature is substantial, is in general less sensitive to temperature. Overall the observed differences in photoperiod response can be broadly associated with the ecological adaptations of different races within the two genepools (Singh, 1988, 1989; Smartt, 1988). This variation is interesting from an evolutionary point of view, but presents a challenge for matching phenology to environment and planting time, and generally for improving common bean production in temperate regions. The existence of two common bean gene pools deriving from independent domestication events is an important characteristic of the species and provides additional variation and challenges for matching and developing varieties for different climatic and planting time conditions and generally for improving temperate bean production.

Similar to other well-known SDP crop species such as rice, maize, and soybean which are excellent model systems for elucidating time to flowering (Ebana et al., 2011; Xu et al., 2012; Lu et al., 2016), the broad adaptability of common bean to a wide range of latitudes depends on natural genetic variation for flowering time, and a number of major genes and/or quantitative trait loci (QTL) have been described (Koinange et al., 1996; Tar’an et al., 2002; Blair et al., 2006; Pérez-Vega et al., 2010; Mukeshimana et al., 2014; Kamfwa et al., 2015; González et al., 2016; Moghaddam et al., 2016; Bhakta et al., 2017; Wallach et al., 2018). Bhakta et al. (2017) identified twelve QTL controlling time to flower on chromosomes 1, 3, 4, 6, 7, and 11, and reported interactions with specific environmental factors such as temperature, photoperiod, or solar radiation. This study indicated how different QTL allele combinations may determine desired phenotypes under specific environments. González et al. (2016) detected six QTL on chromosomes 1, 2, 5, 7, 9, and 10, and environment and epistatic interactions in the genetic control of flowering time. This study also suggested that several of the QTL identified might have pleiotropic effects on aspect of vegetative growth. Other recent research based on genome-wide association studies (GWAS) identified thirteen significant associations of flowering time and potential candidate genes on chromosomes 1, 4, 6, 7, and 8 (Moghaddam et al., 2016; Raggi et al., 2019). A large-scale GWAS with 683 common bean accessions (Wu et al., 2020) detected 101 associations of flowering time on all chromosomes aligned with candidate genes, and determined their prevalence across years and north–south geographic clines. These studies collectively illustrate how flowering time can be modulated under long-day (LD) or short-day (SD) photoperiodic conditions, and the importance of this for alignment with production location. However, understanding of the genes involved in the timing of flowering remains relatively limited.

In soybean, flowering time control pathway features a central role for flowering locus T (FT) genes, including both promoters and inhibitors of flowering (Zhai et al., 2014; Zhao et al., 2016; Liu et al., 2018). A key step in photoperiod response is the SD-repression of the E1 gene, a legume-specific gene that inhibits flowering through direct transcriptional regulation of FT homologs (Xu et al., 2015; Cao et al., 2017; Zhu et al., 2019). Other upstream genes such as homologs of the PHYTOCHROME A (PHYA), the maturity genes E3 and E4 (Watanabe et al., 2009), and the circadian clock related genes J, Tof11 and Tof12 (Lu et al., 2017, 2020) appear to act mainly through regulation of E1. Soybean homologs of well-characterized Arabidopsis flowering genes GI and CO also influence flowering time (Watanabe et al., 2011; Cao et al., 2015) but have not been fully integrated into pathway models. These examples indicate the broad conservation of certain central components of photoperiodic flowering but also point to the likely existence of novel genes, unique variations and possible alternative mechanisms responsible for differences between species and between LDP and SDP.

The importance of chromosome 1 controlling time to flowering has been reported in common bean (Gu et al., 1998; Kwak et al., 2008; Pérez-Vega et al., 2010; Repinski et al., 2012; González et al., 2016), and features two linked loci that may both influence this trait. The major photoperiod sensitivity locus *Ppd* (Wallace et al., 1993; Koinange et al., 1996) was recently identified as the red/far-red photoreceptor gene *PHYTOCHROME A3* (*PHYA3*), an ortholog to soybean *E3*, with distinct loss-of-function *PHYA3* mutations present in Andean and Mesoamerican gene pools (Weller et al., 2019). A second major locus, *Fin*, primarily controls shoot determinacy but may also contribute to flowering time control (Weller et al., 2019), and is an ortholog of Arabidopsis *TERMINAL FLOWER1* (*TFL1*) and the soybean *Dt1* locus (Strasser et al., 2009; Hanano and Goto, 2011; Repinski et al., 2012). Beyond these examples, little is currently known about the molecular basis for flowering time control in common bean, and there is still substantial variation in the global germplasm that is unexplained. In fact, among the genetic analyses mentioned above, few have specifically addressed the genetic basis of photoperiod sensitivity. In this study, we conducted a genetic analysis of flowering time and photoperiod response in a recombinant inbred (RI) population between an adapted accession and a photoperiod sensitive landrace of common bean in twelve different LD and SD environments across 6 years. We report two novel major loci on chromosomes 4 and 9, which control flowering under long- and short-daylengths, respectively, and show complex epistatic and environment interactions. Our results build a foundation for breeding of high-yield common bean varieties with optimum adaptation to target environments, and for future detailed molecular analysis of the underlying genes.

## Materials and Methods

### Plant Material

**FIGURE 1:**
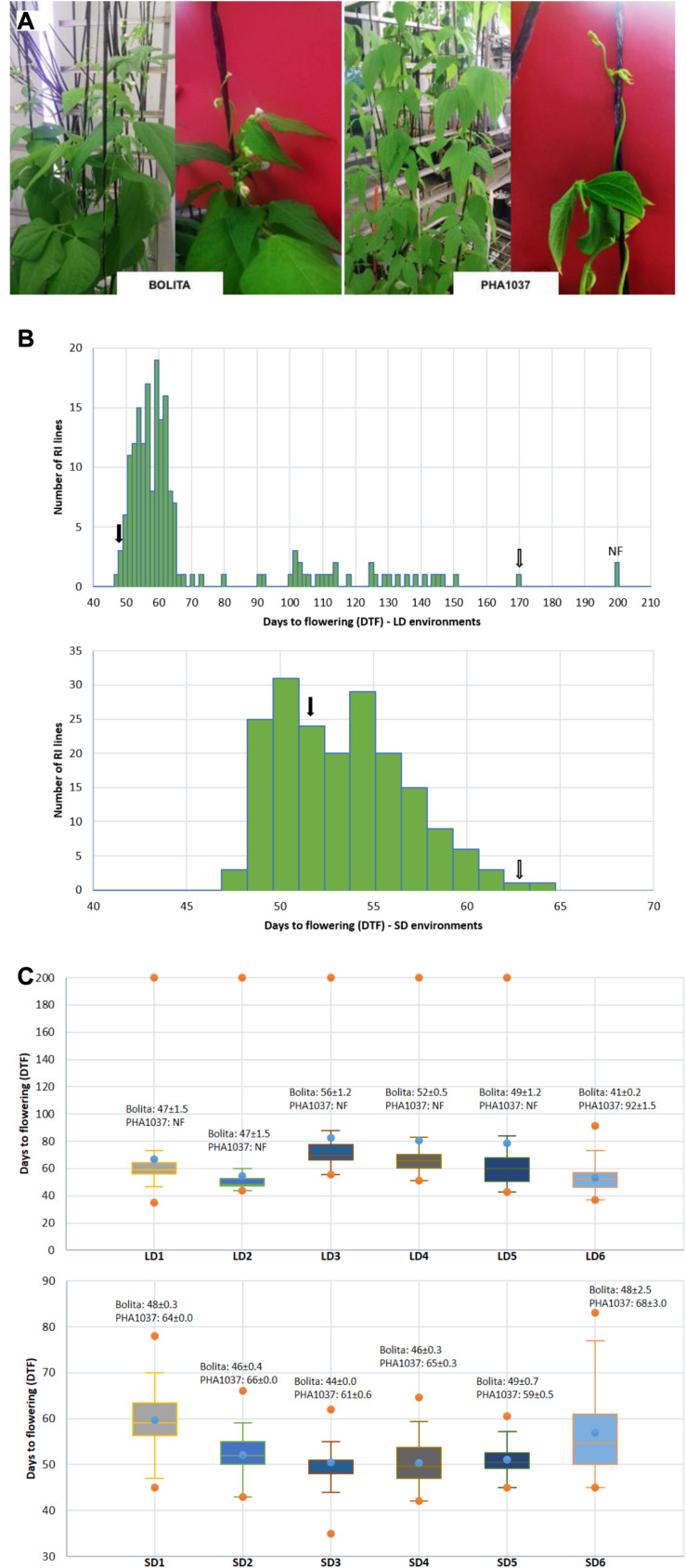
Effect of short- and long-day length (SD and LD) on flowering time in common bean. **(A)** Images illustrating plants of cultivar Bolita and landrace PHA1037 grown under LD conditions at 6 weeks after planting. **(B)** Distribution of days to flowering (DTF) and number of individuals of the RI population under all LD and SD trials; where black and white arrows correspond to Bolita and PHA1037 parents, respectively. **(C)** Comparison of days to flowering (DTF) of RI population in the LD and SD different trials across 6 years. *Y*- and abscissa axes represent DTF and LD or SD trials, respectively. Means and standard errors for Bolita and PHA1037 accessions for each trial are shown, NF = non-flowering (Supplementary Table 2). Specific characteristics of the twelve trials are shown in Table 1.

A RI population named as BN was generated between two Andean accessions, Bolita (PMB0225 MBG code, female parent) and PHA1037 (G23617 CIAT code, male parent). A total of 249 F_2_ seeds of the cross were initially advanced by single seed descent for seven generations, followed by bulk propagation for another three generations, giving rise to 185 (F_2__:__7_) RI lines. Bolita is a large white seeded cultivar from Spain with a type II indeterminate erect growth habit, and PHA1307 is a large-seeded red nuña accession from Bolivia, with a type IV indeterminate climbing growth habit (Figure 1A); according to the nomenclature of Centro Internacional de Agricultura Tropical CIAT (1986), where I = determinate erect or upright, II = indeterminate erect, III = indeterminate prostrate, and IV = indeterminate climbing.

### Experimental Design and Phenotypic Data

The RI population was evaluated from 2009 to 2016 under field and semi-controlled conditions at Northwest Spain (latitude 42°24′N, longitude 8°38′W, and altitude 40 masl) in twelve natural photoperiod trials (Supplementary Figure 1 and Table 1), with a LD and SD environment in each trial year and a range of sowing dates from late-February to late-July (LD; average day length is 13 h 40 min, from the first 100 days after sowing) and from mid-August to late-September (SD; average day length is 10 h 80 min, from the first 100 days after sowing). Climatic variables were downloaded from https://www.meteogalicia.gal and https://www.timeanddate.com. A randomized complete block design with two plants per replication and two replications per line was employed in each environment, where each line was planted in one 3 m-long row, 0.80 m between rows, and a 30,000 plant ha^–1^ of crop density. The semi-controlled SD conditions were conducted under a heated soil greenhouse, with a temperature range between 8 and 14°C (Table 1). Crop management was in accordance with local practices.

**TABLE 1:** Meteorological characteristics of the 12 environments across 6 years.

**Environment**	**Sowing date**	**Maximum temperature (°C)**	**Minimum temperature (°C)**	**Solar radiation (10 kJ/(m^2^día))**	**Daylength average (h:min)**	**Daylength range (h:min)**
LD1	February 20, 2009	19.42	7.55	1669	13:06	10:49–15:03
LD2	March 01, 2011	22.28	10.38	1737	13:27	11:13–15:13
LD3	March 02, 2016	18.59	9.03	1596	13:32	11:18–15:14
LD4	March 15, 2010	19.00	8.30	1680	13:59	11:54–15:17
LD5	April 27, 2015	24.61	13.17	1875	14:51	13:53–14:19
LD6	July 26, 2013	25.04	14.50	1431	12:30	14:39–10:14
SD1	August 12, 2013	23.55	13.59	1296	11:45	14:01–9:36
SD2	August 20, 2009	21.85	12.85	1097	11:24	13:41–9:22
SD3	August 24, 2011	21.53	11.56	1082	11:15	13.32–9:17
SD4	August 26, 2015	20.67	11.72	1015	11:10	13:26–9.15
SD5	September 13, 2016	19.54	9.90	924	10:27	12:34–9:04
SD6	September 21, 2010	16.95	7.87	731	10:12	12:13–9:06

*Data observed during the first 100 days after sowing.*

Days to flowering (DTF) was recorded as days between emergence and the opening of the first flower per line. For plants that did not flower at the end of the LD experiments, DTF was assigned a value of 200 days. To evaluate the temperature effect of each environment, the daily average temperature for 100 days after sowing was measured to quantify the DTF as the growing degree days (GDD). GDD was calculated as = [(T_MAX_ + T_MIN_)/2]–T_BASE_ (McMaster and Wilhelm, 1997), where T_MAX_ and T_MIN_ are the maximum and minimum daily temperatures, respectively; and T_BASE_ is 10°C as the temperature below which growth ceases. If T_MAX_ < T_BASE_ then T_MAX_ = T_BASE_, and if T_MIN_ < T_BASE_ then T_MIN_ = T_BASE_.

To evaluate the effect of vegetative growth on time to flowering, the internode length (IL) was measured by selecting an internode from the midpoint along the main stem of the plant and recording its length in centimeters. In addition, we measured the number of pods per plant (PP) and growth habit (GH), which was scored following the nomenclature of Centro Internacional de Agricultura Tropical CIAT (1986).

To evaluate the effect of photoperiod, the DTF difference between LD and SD in each year was determined (2009 = LD1 vs. SD2; 2010 = LD4 vs. SD6; 2011 = LD2 vs. SD3; 2013 = LD6 vs. SD1, 2015 = LD5 vs. SD4; and 2016 = LD3 vs. SD5). The DTF LD vs. SD difference was quantified as Photoperiod Response Index (PRI = DTF_LD_-DTF_SD_; Cuevas et al., 2016), Percentage of Photoperiod Sensibility (PS = (DTF_LD_–DTF_SD_)/DTF_LD_ × 100; Jiang et al., 2014), where values <30 and >50% are classified as insensitive and high sensitivity to photoperiod, respectively, and the Relative Response to Photoperiod (RRP) (RRP = 1–(R_LD_/R_SD_), R = 1/(DTF-DTE), where DTE is days from sowing to seedling emergence; White and Laing, 1989), and when no flowering occurred in LD, R_LD_ = 0 and RRP = 1 or highly photoperiod-sensitive. A photoperiod response classification (CLASS) was determined as the mean number of days delay in flowering due to photoperiod according to a scale of 1–8 (White and Laing, 1989), as follows: 1 = 0 to 3, 2 = 4 to 10, 3 = 11 to 19, 4 = 20 to 39, 5 = 40 to 59, 6 = 60 to 79, 7 = 80 to 99, and 8 = over 100, days delay in flowering. Grouping response classes 1 and 2 were classified as day-neutral, 3 and 4 as intermediate, and 5–8 as sensitive.

### Analysis of Phenotype Data

Statistical analysis was performed using SAS09 (Institute, Inc., 9.04, Cary, NC, United States). The analysis of variance was conducted with the PROC MIXED procedure. Parents were considered fixed genotypes and RI lines were considered random effects. Replications, environments, and environment-by-genotype interaction were also considered random effects. In addition, the relationship between GDD of each LD and SD was tested by using Pearson’s correlation coefficient (r^2^). Variance components were estimated using the PROC VARCOMP procedure (all effects as random). Heritability (h^2^) was calculated as [σ^2^_G_/(σ^2^_G_ + σ^2^_E_/r)] × 100%, where σ^2^_G_ is genotypic variance, σ^2^_E_ is error variance, and r is the number of replications (Holland et al., 2003).

### QTL Detection in Different Environments

A genetic linkage map for the BN RI population was initially developed by Yuste-Lisbona et al. (2012), and substantially supplemented with new markers by González et al. (2015) and in this study (Supplementary Table 1). Markers were added to the map with the JoinMap^®^ 4.1 software (Van Ooijen, 2006) by using a regression mapping algorithm. A minimum logarithm of odds ratio (LOD) score of 6.0 and a maximum recombination fraction of 0.3 were set as the linkage threshold for grouping markers. Physical positions were identified by using nucleotide sequences of 200 markers as queries for BLASTN against the chromosome-scale *P. vulgaris* V2.1 genome assembly, available in the Phytozome database^1^. Spearman correlation coefficients were calculated to assess the collinearity between the genetic marker positions (in centimorgans, cM) against their physical positions (in megabases, Mb).

A multi-environment QTL analysis was performed by using QTL Network 2.0 software (Yang et al., 2008) to identify putative main QTL (QTL with significant genetic main effects), epistatic QTL and their environment interactions effects (QTL × environment, QE; and epistatic QTL × environment, epistatic QE), according to a mixed-model based composite interval mapping method (MCIM). An experimental-wise significance level of *P* < 0.05 was designated for candidate interval selection, putative QTL detection and QTL effect. Both testing and filtration window size were set at 10 cM, with a walk speed of 1 cM. The critical *F* value to declare putative QTL was determined by a 1000 permutation test at the confidence level of 95%. The identified QTL were named by the trait and chromosome number. A given QTL was defined as major when it was identified in at least one environment explaining >20% phenotypic variation, or in at least two environments explaining >10% phenotypic variation.

In an attempt to summarize the effects of the climatic variables (maximum temperature, solar radiation, and daylength) on covariance of DTF, a principal component analysis (PCA) was done across LD and SD environments. Pearson correlations between the variables were computed and thereafter PCA was implemented by using the XLSTAT6.0 (Addinsoft, Inc., New York, NY, United States) to obtain PCs for each genotype. Pearson product-moment correlation coefficients rather than covariances were used because measures were not in comparable scales. Any PC with an eigenvalue <1 was considered to be noise and eliminated. PCs were later used as multivariate quantitative phenotypes subject to conventional genetic analysis of measured phenotypes. A genome-wide detection for QTL influencing PCs was done using QTL Network 2.0 software (Yang et al., 2008).

The physical intervals harboring major QTL were selected for inferring potential candidate genes. All genes included in each significant QTL interval along with their Arabidopsis putative homologs were identified with the PhytoMine interface of Phytozome (Goodstein et al., 2012). Gene information was obtained from the National Center for Biotechnology Information (NCBI). PCR from genomic DNA was used to amplify the full-length candidate genes underlying the QTL DTF-1.4 (Phvul.001G221100 and Phvul.001G232900), DTF-4.1 (Phvul.004G046601 and Phvul.004G037600), DTF-9.1 (Phvul.009G013900 and Phvul.009G018700), DTF-9.4 (Phvul.009G204600), and DTF-9.5 (Phvul.009G203400). Primer set used for PCR amplification experiments are indicated in Supplementary Table 2. PCR products from Bolita and PHA1037 accessions were sequenced by conventional Sanger technology using BigDye^®^ Terminator v3.1 chemistry and the Applied Biosystems^TM^ 3500 Series Genetic Analyzer. Sequence analysis and alignments were performed using Geneious software^2^.

## Results

### Genetic Architecture of Flowering Time and Photoperiod Response

To gain a better understanding of the molecular mechanisms underlying common bean flowering time and photoperiod response, we evaluated a RI population derived from a biparental cross between Bolita, a photoperiod-insensitive early flowering cultivar from Spain, and PHA1037, a landrace from Bolivia with a strong photoperiod response similar to wild accessions (Figure 1A). This population was evaluated in twelve different trials across 6 years, with a LD and SD environment in each trial year, and a range of sowing dates from late-February to late-July (LD) and from mid-August to late-September (SD) (Supplementary Table 3). Days to flowering was also quantified as the number of growing degree days to flowering, in order to consider temperature differences among environments. Photoperiod response for each RI line was measured as the difference in mean DTF between LD and SD within each year.

Days to flowering of the two parents, Bolita and PHA1037, was significantly different (*P* < 0.001) in all environments with increased flowering time or non-flowering for PHA1037 in LD (Figure 1B). A significant effect of RI genotype and environment was also detected for DTF in each year (for full ANOVA results, see Supplementary Table 4). DTF showed a higher *h*^2^ value (>85%) and genetic variance in LD environments compared to SD (*h*^2^ ranged from 43 to 86%) (Table 2 and Supplementary Table 4). We also detected a weak positive correlation (*r*^2^ = 0.29^∗∗∗^) for DTF between LD and SD (Table 2), indicating the possible involvement of a genetic component independent of daylength. The RI population segregated widely for DTF in LD (Figures 1B,C, spanning 7 to >20 weeks). In SD, DTF range was much narrower (6–10 weeks in most years) and displayed approximately a normal distribution with some evidence of bimodality or skewing toward earliness (Figures 1B,C). The longer daylength and higher solar radiation under LD (Table 1 and Supplementary Figure 1) was associated with an effect of delaying time to flowering (LD and SD mean DTF 69 ± 5.4 and 53 ± 1.7 days, respectively).

**TABLE 2:** Analysis of variance results partitioning variation for days to flowering (DTF) (for full ANOVA see Supplementary Table 2) and associated quantitative genetic parameters of variance components (V_G_ and V_P_) and heritability (h^2^) for RI lines growing in Short (SD) and Long-Day (LD) environments (E).

**Trait**	**SD environments**					**LD environments**					**Line × E**	**Across SD–LD correlation**
	**Line**	**Line × E**	**V_G_**	**V_p_**	***h*^2^**	**Line**	**Line × E**	**V_G_**	**V_p_**	***h*^2^**		
DTF	***	***	7.47	33.99	0.22 ± 0.03	***	***	577.35	1119.43	0.52 ± 0.03	***	0.29***

From an additional ANOVA using PROC MIXED (SAS Institute Inc., V. 9.4, Cary, NC, United States), the line × environment interaction (for full ANOVA see Supplementary Table 2) tests for evidence of genetic variation in flowering time across environments. *** significant at the 0.05, 0.01, and 0.001 probability levels, respectively. *h*^2^ with their standard errors were estimated by restricted maximum likelihood (REML) option of the PROC MIXED and IML (Holland et al., 2003; Holland, 2006). Phenotypic Pearson correlation coefficients among traits were implemented using PROC CORR across the environments.

**FIGURE 2:**
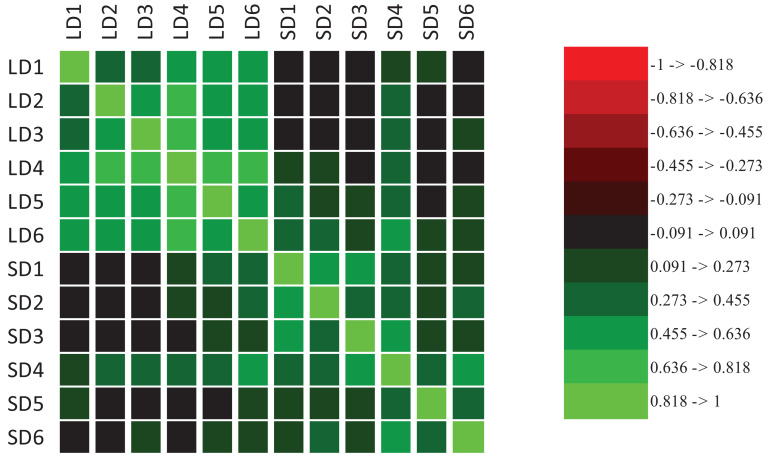
Correlation heat map of time to flowering expressed as growing degree days (GDD) between LD and SD environments.

Evidence of transgressive segregation was detected in both LD and SD; that is, the range of trait expression among RI lines exceeded that of the parental lines (for full distribution results per each environment see Supplementary Figure 2). When RI plants were grown under LD, from the beginning of March to late-July (LD3-6), a trend toward earlier flowering with increasing temperature was observed (Supplementary Figures 1, 3). Mean DTF ranged from 83 down to 53 days, from lowest to highest mean temperature for LD3-6 (Supplementary Table 4). A difference was also seen in the appearance of non-flowering plants, which occurred in a proportion of 1 to 15% for LD1-5, but were not seen in LD6, where all plants flowered by 92 day (Supplementary Figure 2), which is eventually a SD after 8 weeks from sowing (Supplementary Figure 1). Flowering time was positively correlated among environments, and the correlation coefficients were higher for comparisons within LD or SD than for comparisons between LD and SD (Figure 2). These results indicate that a strong genetic component underlies variation for DTF in this population, especially under LD, and emphasize that DTF is a complex trait that displays pronounced genotype by environment interaction.

**FIGURE 3:**
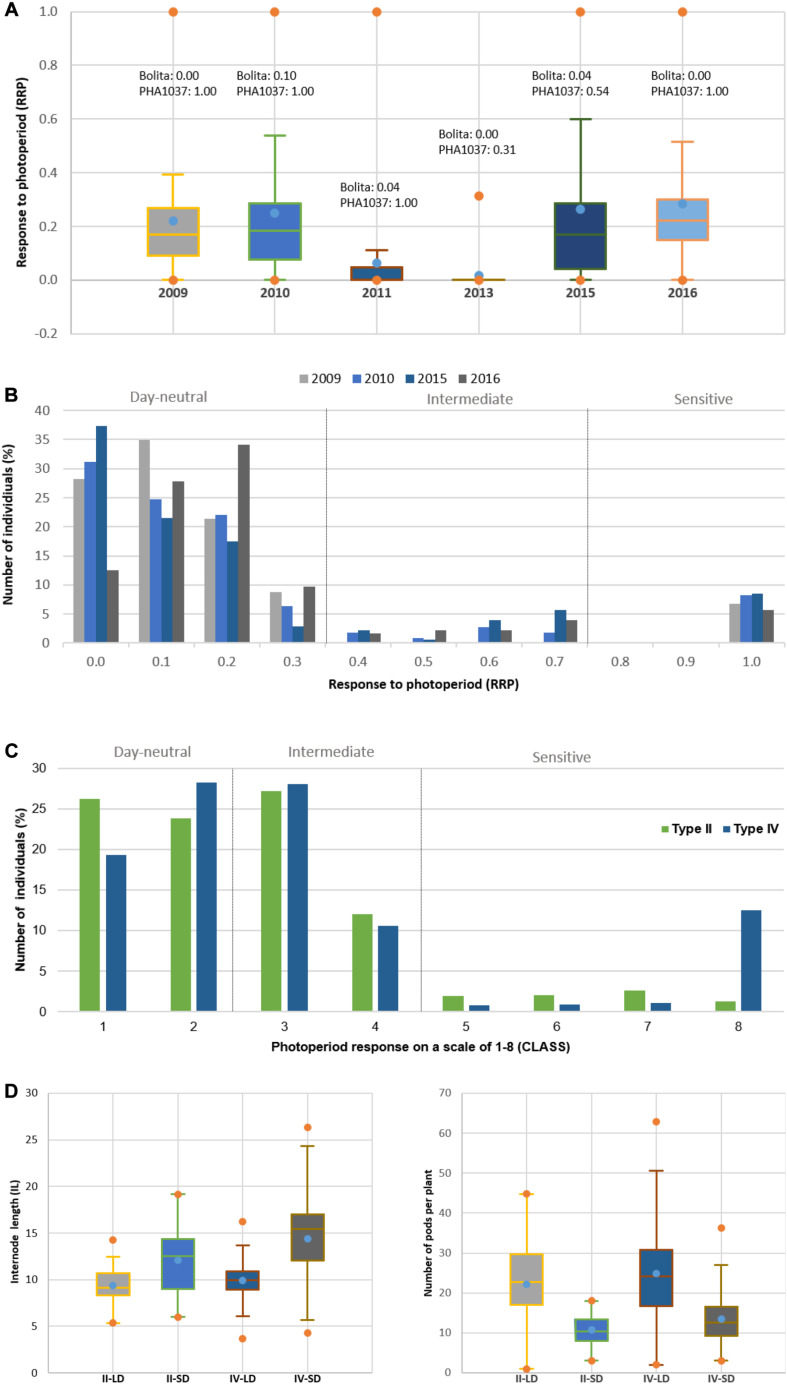
Effect on photoperiod response in common bean. **(A)** Comparison of the Relative Response to Photoperiod (RPP) for the RI population across the six trial years. *Y*-axis represents RRP and *X*-axis different years. RRP was measured as a relative change in rate of flowering under LD and SD environments, where values of 0 indicate a day-neutral response and values of 1 indicate maximum response to photoperiod. **(B)** Distribution of RRP and percentage of individuals of the RI population across the trial years (2011 and 2013 years are not included due to the low variation observed). Three groups of responses: day-neutral group (RRP = 0–0.2), intermediate group (RRP = 0.3–0.7), sensitivity group (RRP ≥ 0.7). **(C)** Distribution of the photoperiod response on a scale of 1–8 (CLASS) and percentage of individuals of the RI population for the type II and type IV growth habits across 2009 to 2016 trail years. Grouping response classes 1 and 2 were classified as day-neutral, 3 and 4 as intermediate, and 5–8 as sensitive. **(D)** Variation of internode length (IL) and number of pods per plant (PP) for type II and type IV RI lines across LD and SD environments. Bolita and PHA1037 parents show an indeterminate type II and IV growth habit, respectively.

**FIGURE 4:**
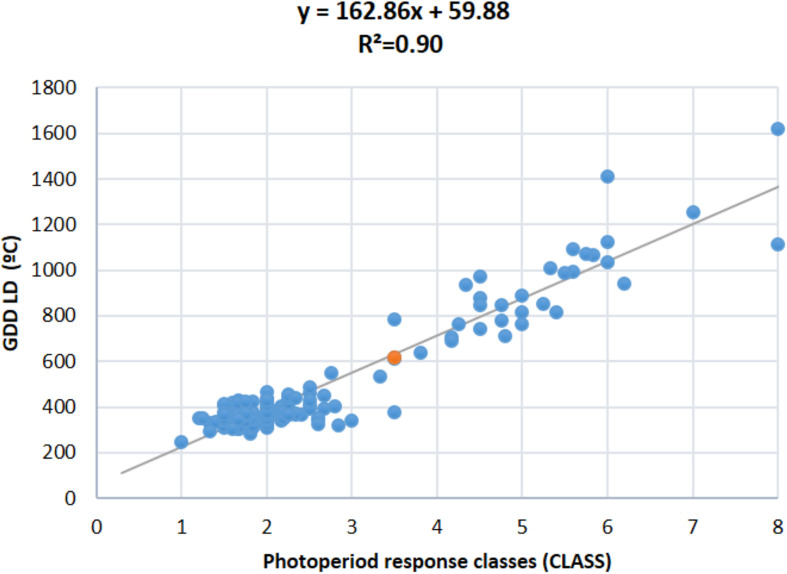
Regression of observed photoperiod response classes (CLASS, scale 1–8) and flowering time expressed as growing degree days (GDD) under LD.

In general, the trends and relationships seen for DTF are also reflected in the photoperiod response (quantified as PRI, PS, RRP, and CLASS traits), where some of the most strongly photoperiod-sensitive lines failed to flower or showed delayed flowering in LD in all trial years, except in 2011 and 2013 which showed lower variation and were discarded (Figure 3A and Supplementary Figure 4). When lines were classified for photoperiod response (White and Laing, 1989), 25 to 55% were day-neutral (classes 1 and 2, <10 days delay in flowering) in 3 years (2009, 2010, and 2015, mean 39%), but only 11% in 2016. Less than 26% of the RI lines did not flower under LD (Supplementary Figure 4). By re-expressing the data in terms of RRP, three groups of responses were identified. A low response or day-neutral group (RRP = 0–0.2, 13–37% genotypes, mean 26%), an intermediate group (RRP = 0.3–0.7, 1–10% genotypes, mean 2%), and a maximum sensitivity group (RRP = 1, 6–9% genotypes, mean 7%) (Figure 3B). A strong association was found between photoperiod response classes and GDD under LD (*r*^2^ = 0.90; Figure 4), which indicates that genomic regions associated with these traits are likely to be similar.

We also observed evidence of a relationship between photoperiod sensitivity and vegetative development. Whereas day-neutral genotypes (classes 1 and 2) occurred at equivalent frequencies of 19 to 28% in both types II and IV growth habits, higher frequency of highly sensitive genotypes (class 8) was seen among RI lines with type IV (13%) compared to type II (1%) (Figure 3C). Internode length was also influenced by the growth habit and the environment daylength (Figure 3D), because the plant climbing ability or length of the main stem is determined by the number of internodes and their length (Checa et al., 2006). A reduction in the elongation of internode was observed under LD (types II and IV, mean = 9 cm) relative to SD (types II and IV, mean = 12 and 14 cm, respectively). However, pod number increased during LD (Figure 3D) as the DTF duration in this phase is lengthened (Egli and Bruening, 2000; Kantolic and Slafer, 2005). The results show that longer daylengths enhance delay of flowering through the activity of genes that control the response to photoperiod, contributing to the vegetative development of the plant, and support the reliability and strength of the phenotypic data for mapping QTL.

### Identification of QTL and Epistatic Interactions for Flowering Time Under Long-Day and Short-Day Conditions

In order to analyze the genetic control of the traits measured, we saturated the genetic map for the RI population published in 2015 with 54 new markers, including 15 specifics for flowering-related genes. The genotypic ratios of a relatively large proportion of markers deviated significantly from the expected Mendelian ratios (Supplementary Table 1). However, distorted markers were not excluded from the mapping analysis, because segregation distortion is expected to be prevalent in a RIL population and omitting such markers would result in low coverage in many regions of the genetic map. The total length of the genetic map is 879 cM, with an average genetic distance between adjacent markers of 3.11 cM, and a maximum distance between consecutive markers of 28.43 cM. The mean ratio between physical and genetic distance is 617.78 kb/cM (Table 3). Collinearity of the map was evaluated against the *P. vulgaris V2* genome assembly using nucleotide sequences from 200 markers, with a clear relationship between marker positions on the genetic and physical map, providing a solid basis for the QTL analysis. Spearman’s correlation coefficients ranged from 0.66 for chromosome 2 to 0.99 for chromosomes 6 and 11 (Supplementary Figure 5).

**TABLE 3:** Number of identified QTL per chromosome for time to flowering and photoperiod response in the LD and SD environments and across 6 years.

**Chromosome**	**Ratio kb/cM**	**Time to flowering (DTF, GGD)^a^**				**Photoperiod response (PRI, PS, RRP, CLASS)^b^**	
		**LD**		**SD**		**Years**	
		**Main QTL**	**Epistatic QTL**	**Main QTL**	**Epistatic QTL**	**Main QTL**	**Epistatic QTL**
Chromosome 1	303.76	2	1	3		1	2
Chromosome 2	722.71				1		7
Chromosome 3	470.93		1				1
Chromosome 4	794.34	2	5	3		6	11
Chromosome 5	701.66		2	2	1	4	2
Chromosome 6	708.43						2
Chromosome 7	524.24			3	1	1	
Chromosome 8	933.31			1	1		6
Chromosome 9	374.96	2	3	5		6	8
Chromosome 10	569.45		1	2			1
Chromosome 11	691.81						

*Days to flowering = DTF, growing degree days = GDD; photoperiod response index = PRI, percentage of photoperiod sensitivity = PS, relative Response to Photoperiod = RRP, and photoperiod response on a scale of 1-8 = CLASS.*

*^*a*^Number of main and epistatic QTL detected in SD and LD environments.*

*^*b*^Number of main and epistatic QTL detected across 6 years.*

**FIGURE 5:**
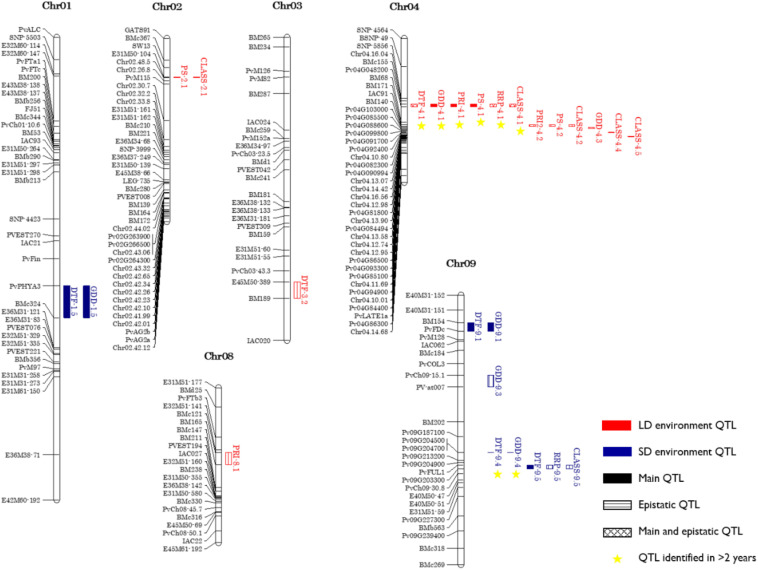
Genetic linkage map showing main QTL and epistatic QTL explaining >10% of the phenotypic variation at least in one environment. Names of markers are shown on the left. QTL are depicted as vertical bars to the right of the chromosomes.

Combining the genetic linkage map with the phenotypic data for flowering time in each of the LD and SD, a total of 19 main QTL were detected, 9 for DTF and 10 for GDD (Table 3 and Supplementary Tables 3, 5). Fourteen of these QTL mapped to the same genomic regions on chromosomes 1, 4, 5, 7, 9, and 10, while the remaining 5 QTL were specific to each DTF or GDD traits (DTF-1.3, DTF-9.5, GDD-4.6, GDD-7.1, and GDD-8.1). A total of 18 QTL for four photoperiod response traits were identified in nine genomic regions (four for PRI, five for PS, three for RRP, and six for CLASS traits) (Table 3 and Supplementary Table 5). Three of these nine genomic regions bearing QTL for more than one photoperiod response trait and the remaining six contained QTL for a single trait (PRI-1.1, PS-9.2, PS-9.4, CLASS-4.4, CLASS-4.5, and CLASS-7.2). It is noteworthy that QTL alleles from PHA1037 parent did not delay always flowering, as 7 of the 37 detected QTL (CLASS-4.4, GDD-4.6, GDD-7.1, DTF-7.2, GDD-7.2, CLASS-7.2, and PS-9.2) had negative additive values, which indicates that alleles from PHA1037 parent also contribute to reduce time to flowering. The DTF and GDD QTL accounted for 28 and 10% on average of the phenotypic variation in the population across LD and SD, respectively; while QTL for photoperiod response traits explained an average of 25, 22, 26, and 24% of the phenotypic variation for PRI, PS, RRP, and CLASS, respectively (Supplementary Table 5). Consistent with the correlation observed between photoperiod response and LD flowering described above (Figure 4), the main QTL associated with photoperiod response tended to co-localize to those QTL detected for flowering time under LD conditions (Figure 5). Furthermore, a similar magnitude of the main QTL effects for photoperiod response and LD flowering time was observed (Supplementary Table 5).

Among all main QTL, 17 genomic regions were identified, representing from 1 (chromosomes 8 and 10) to 4 (chromosomes 4 and 9) regions per chromosome (Supplementary Table 5 and Figure 5). The genomic regions on chromosomes 4 and 9 accounted an average of 38 and 23% of mean phenotypic variation for DTF in LD and SD, respectively; whereas the remaining QTL had a smaller but significant contribution to the total phenotypic variation. At both loci, the positive allele effect (delayed flowering) was conferred by PHA1037. This result suggests the importance of these genomic regions in shaping the genetic architecture responsible for the measured variation in photoperiod response and flowering time in LD and SD. The major QTL DTF-4.1 on chromosome 4 was detected in five out of the six LD environments (and in LD3 as an epistatic QTL; Supplementary Table 6) and co-located with QTL for GDD and photoperiod response traits in all trial years (Figure 5). The DTF-4.1 QTL explained 55 and 62% of the total phenotypic variance for DTF in the LD4 and LD5 environments, respectively, which had the higher daylength (average 13:59 and 14:51 h, respectively), while it accounted for 22, 19, and 30% of the total phenotypic variation in LD1, LD2 (average 13:06 and 13:27 h, respectively) and LD6 (daylength drop <12 h after 8 weeks from sowing), respectively (Table 1, Supplementary Figure 1, and Supplementary Table 5). The DTF-4.1 QTL was also detected in SD1 and SD4 environments but explained a small portion of the phenotypic variation (less than 9%) (Supplementary Table 5). Taken together these results indicate that DTF-4.1 QTL may have a primary role in controlling photoperiod response.

The second most significant main QTL for DTF (on chromosome 9; DTF-9.4) explained a higher phenotypic variation under the lowest daylength environment (*R*^2^ = 14 and 32% in SD3 and SD4, with an average of 11:15 and 11:10 h of daylength, respectively). The DTF-9.4 QTL co-located with the GDD-9.4 QTL, which accounted up to 32% of the phenotypic variance under SD conditions (*R*^2^ = 9, 12, and 32% in SD1, SD3, and SD4, respectively). This QTL was also detected in LD5 although explaining a percentage of the phenotypic variance minor than 2% (Figure 5 and Supplementary Table 5). A third QTL for DTF located on chromosome 1, DTF-1.4, had a greater influence on flowering in SD (*R*^2^ = 10% in SD3) compared to LD (LD6, *R*^2^ = 5%), and co-located with the GDD-1.4 QTL, accounting up to 11% of the phenotypic variance in SD (*R*^2^ = 10 and 11% in SD2 and SD3, respectively), while it explained 6% in LD6 condition Supplementary Table 5). Other QTL that mapped to Chr09 (DTF-9.1) was only detected in SD6, where it explained 16% of the phenotypic variation, and did not co-located with QTL for GDD. It is interesting to note that QTL for photoperiod response traits were not detected in the genomic regions where these three QTL, DTF-9.4, DTF-1.4, and DTF-9.1, were located. Finally, the genomic region harboring the DTF-9.5 QTL explained up to 14% of the phenotypic variance in SD (*R*^2^ = 9 and 14% in SD1 and SD6, respectively) but it only reached 2% in LD5. The DTF-9.5 QTL co-located with several QTL for photoperiod response traits (PRI-9.5, PS-9.5, RRP-9.5, and CLASS-9.5); however, these QTL explained <5% of the phenotypic variation (Supplementary Table 5).

We explored our data further in a genome-wide epistatic interaction analysis in order to evaluate how relationships among genomic regions affect flowering time and photoperiod response (Supplementary Table 6). A total of 34 interactions involving 53 epistatic QTL were detected for flowering time in LD (DTF and GDD) and photoperiod response (PRI, PS, RRP, and CLASS) traits, whereas only two interactions among four epistatic QTL were found in SD, both for DTF. The highest number of interactions were found for epistatic QTL located on chromosomes 4 and 9 (Table 3). Seventeen of the epistatic QTL identified were previously detected as main QTL, which indicated that these QTL not only participated in epistatic interactions, but they also had an individual effect. The estimated additive values of epistatic interactions were negative for 44% of the interactions detected, indicating that alleles from Bolita also play a role in delaying flowering. Interestingly, epistatic interactions involving the genomic region on chromosome, 4 where the main QTL DTF-4.1, GDD-4.1, PRI-4.1, PS-4.1, RRP-4.1, and CLASS-4.1 were located, explained the major percentage of phenotypic variance, reaching values up to 15 and 22% for flowering time and photoperiod traits, respectively (Supplementary Table 6).

In addition to the main and epistatic QTL identified, environment interaction effects (QTL × Environment, QE; and epistatic QTL × Environment, epistatic QE) were detected (Supplementary Table 7) as a means of accounting for inconsistent detection of QTL between environments. The QE interaction analysis showed significant effects under LD and SD for four flowering time QTL, while significant QE were found in 16 QTL for photoperiod response traits. The impact of the additive × environment interaction effect (*ae*) was different across certain LD and SD environments. Among these, we highlighted the major loci DTF-4.1 and DTF-9.4, explaining 14 and 6% of the phenotypic variance under LD and SD, respectively. Remarkably, at the QTL DTF-4.1, alleles from PHA1037 could delay flowering through significant and positive *ae* effects in the longest daylength LD4-5, but also alleles from Bolita could reduce time to flowering through significant and negative *ae* effects in the shortest daylength of LD1, LD2, and LD6. The DTF-9.4 QTL displayed similar behavior, with a positive and negative *ae* effect in SD6 and SD5, respectively. The instability of these QTL was inferred to be caused by significant *ae* effects and confirm that PHA1037 alleles delayed flowering under the longest daylength environments.

Given that flowering time is a complex polygenic trait, epistatic QTL and their environment interactions may have significant effects on the phenotypic values. Therefore, a two-dimensional genome scan was undertaken for multi-environment QTL analysis which showed that the epistasis × environment interaction effect (*aae*) was an important component of the QE interaction effects. Thus, a total of 32 epistatic QTL involved in 17 interactions were detected for flowering time and photoperiod response traits in different environments (Supplementary Table 8).

**FIGURE 6:**
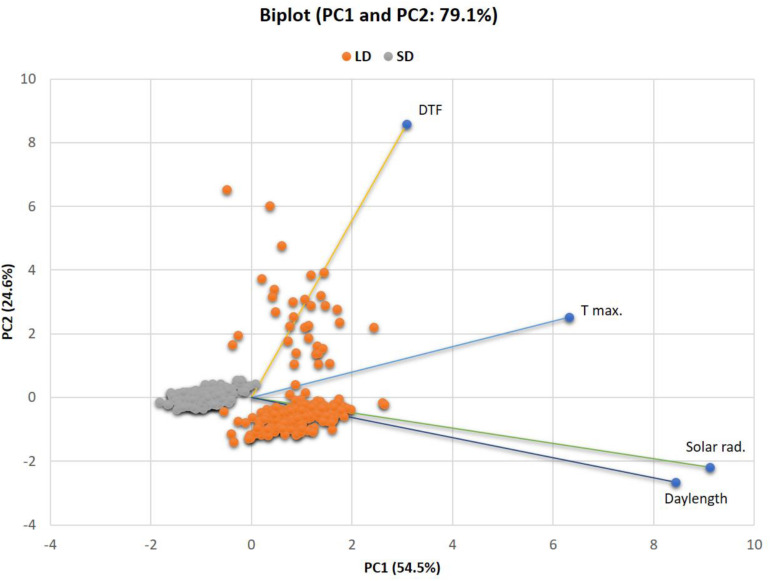
PCA biplot for the relatedness of variables and environments and showing PC values for QTL analysis in LD and SD environments. PC1 and PC2 values for each line are plotted as points and PC1 and PC2 loadings of each variable are indicated by lines. The percent of total variation explained by each PC is labeled on the axes. PC1: first principal component, PC2: second principal component.

A PCA methodology was employed to provide an improved estimate across different LD and SD environments for the genetic effect on phenotypic variance for multi-variable loci. The PCA Biplot (Figure 6) of the climatic variables and DTF across LD and SD showed that together, the PC1 and PC2 accounted for 79.1% (54.5 and 24.6%, respectively) of the total variance in the original traits. The three climatic variables showed high positive (>0.50) correlations with PC1 whereas only DTF was highly correlated with PC2. These results suggest that phenotype PC1 is a composite of the variables daylength, solar radiation, and maximum temperature in descending order of importance, whereas PC2 reflects mainly DTF. Both of these first two PCs were highly responsive the environment, as values for LD environments cluster together separately from those for SD.

In order to determine genetic effects on these components, the calculated values for PC1 and PC2 were used as traits for QTL analysis across all LD and SD. The significant QTL per PC value are summarized in Table 4. A significant putative QTL for PC1 was located on chromosome 1 (*PvALC* and SNP-5503 markers), which contribute to 2% of the phenotypic variance, and was not detected previously by using single-element traits. The estimated additive effect for this QTL was negative and positive for LD and SD, respectively, with alleles from PHA1037 associated with a flowering delay under SD, and those from Bolita with a delay under LD. The other three PC QTL were associated with PC2 and located on chromosomes 4, 8, and 9. For these, two PC2 QTL, PC2-4.1 and PC2-9.5, were observed previously as main QTL with a particular environment. The PC2-4.1 QTL explained 24% of the phenotypic variance and had opposite ae interaction values at LD (positive) and SD (negative). Therefore, alleles from PHA1037 would promote a flowering delay under LD whereas alleles from Bolita would do so under SD. This QTL for a multi-variable PC was as the strongest detected by using the single-variable approach, consistent with the expectation that flowering time in general is influenced by multiple variables, where LD flowering primarily reflects the response to daylength. The identification of both unique and previously observed QTL through this multivariate approach demonstrates the value of working with trait covariance as well as the component traits, and supports that climatic factors and flowering are mechanistically interrelated and consistent with the results of QE interaction observed.

**TABLE 4:** Main QTL and QTL × Environment (QE) effects for PC values using a multi-environment analysis.

**QTL**	**Position in cM^a^**	**Chr.**	**Marker interval**	**F (F threshold)^b^**	**R^2^ (a)^c^**	**A^d^**	**QE AE^e^**		**R^2^ (ae)^f^**
							**LD**	**SD**	
PC1-1.12	0.00–8.28	1	PvALC-SNP-5503	11.42 (7.43)	2.37	−0.11***	−0.12***	0.12***	3.48
PC2-4.1	24.02–25.09	4	BMc155-Pv04G048200	38.56 (7.25)	23.71	0.88***	0.90***	−0.85***	22.92
PC2-8.8	39.54–39.84	8	IAC027-E32M51-160	38.56 (7.25)	1.23	0.11***			
PC2-9.5	57.57–57.57	9	Pv09G204500-Pv09G204700	12.40 (7.25)	0.93	0.28***			

^*a*^Estimated confidence interval of QTL position in cM (Kosambi, 1943). ^*b*^*F* values of significance of each QTL. The critical *F*-value was determined by a permutation test of 1000 repetitions at the confidence level of 95% (Churchill and Doerge, 1994). ^*c*^Percentage of the phenotypic variation explained by additive effects. ^*d*^Estimated additive effect. Positive values indicate that alleles from PHA1037 increase the trait value, and negative values indicate that positive effect is due to the presence of the alleles from Bolita. Experiment-wide *P* value. *** *P* ≤ 0.001. ^*e*^Predicted additive by environment interaction effect. The meaning of sign values is described in the footnote ^*d*^. ^*f*^Percentage of the phenotypic variation explained by additive × environment interaction effect.

### Candidate Gene Identification Based on Flowering Time and Photoperiod Response QTL Analysis

Potential candidate genes underlaying the major QTL were investigated based on the function of their putative homologs in Arabidopsis and other legumes. Scanning of the DTF-1.4 QTL region, flanked by the markers *PvPHYA3* (47.64 Mb) and BMc324 (49.04 Mb) (Figure 5 and Supplementary Table 9), showed the presence of 160 annotated genes. Among these, the gene ID Phvul.001G221100 is ortholog to *PHYA* in Arabidopsis and located at 47.64 Mb, and the gene ID Phvul.001G232900 is ortholog to actin-related proteins (ARP5) and located at 48.67 Mb, which seems to be associated to photoperiod (Supplementary Table 9).

Of the total of 129 genes annotated in the 4.11–5.86 Mb region of the DTF-4.1, flanked by BMc155 and Pv04G048200 (Figure 5), two genes were found to be related to flowering photoperiod and circadian clock and meristem development (Supplementary Table 9). The closest genes to this marker interval were the homologs to *COL2*-*like* (Phvul.004G046601) and *RECEPTOR-LIKE PROTEIN KINASE 2* (*RPK2*) (Phvul.004G037600), which were found to be located about 5.65 and 4.36 Mb, respectively.

The genomic region of the DTF-9.1 (1.86–3.56 Mb, 86 genes annotated), flanked by BM154 and PvFDc (Figure 5), includes the gene ID Phvul.009G013900, an ortholog to *APETALA3* (*AP3*), and Phvul.009G018700, a homolog to *FLOWERING LOCUS D* (*FD*) and ortholog to *VEGETATIVE2* (*VEG2*) in pea (Sussmilch et al., 2015). One of the only three genes annotated in the genomic region of DTF-9.4 (30.99–31.06 Mb) is ortholog to *E1* in soybean, a major gene associated with flowering time and maturity (Xia et al., 2012). Finally, one of the only two genes annotated in the region of the DTF-9.5 (30.86–30.84 Mb) is ortholog to *AGAMOUS*-*like 8* (*AGL8*), which is negatively regulated by *APETALA1* (*AP1*) and is involved in the positive regulation of flower development and inflorescence meristem identity (Mandel and Yanofsky, 1995).

To further explore the potential relevance of the selected candidate genes, genomic DNA from Bolita and PHA1037 parents was used to amplify their full-length sequences. SNP polymorphisms were identified in the exon’s coding sequence of candidate genes underlying DTF-1.4 QTL (Supplementary Table 10). Bolita carried a conservative substitution of a residue (Gly-1066-Ser), typical of insensitive Andean accessions, in the *PHYA3* gene (Phvul.001G221100). In addition, two SNPs were identified in the homolog of the Arabidopsis *ARP5* gene, Phvul.001G232900 (Supplementary Table 10), although only one of them was polymorphic between the two parental lines, which lead to non-synonymous substitution (Glu-494-Val).

With respect to candidate genes for DTF4.1 QTL, four SNP located on the second intron of *PvRPK2* (Phvul.004G037600) were revealed by sequence analysis in both parents, compared to the common bean reference genome; while one SNP located on the 5′ untranslated region was found in *PvCOL2* (Phvul.004G046601) between Bolita and PHA1037 (Supplementary Table 10).

For the DTF9.1 QTL, no differences in *PvAP3* nucleotide sequence were found in *PvAP3* (Phvul.009G013900). In addition, three SNP were detected on the second exon of *PvFDc* (Phvul.009G018700), with two of them which were polymorphic between both parents, and the third SNP showed the same allele in Bolita and PHA1037 (Supplementary Table 10). Regarding the candidate gene for the DTF9.4 QTL, a 38 bp deletion was found in Bolita, 29 bp downstream of the *PvE1* (Phvul.009G204600) stop codon (Supplementary Table 10). Finally, a single InDel was detected on the sixth exon of *PvFUL1* (Phvul.009G203400), the candidate gene for the DTF9.5 QTL (Supplementary Table 10).

## Discussion

Common bean underwent a strong photoperiod response adaptation to flowering time when introduced into Europe and other high-latitude regions (Gepts and Debouck, 1991). Knowledge of the genetic pathways controlling flowering time and how they have been modified to reduce photoperiod sensitivity is advantageous in breeding for promoting yield in temperate regions. The genetics that underlie flowering time variation, its heterogeneity in different LD and SD environments (various growing seasons, temperature regimes, solar radiation and daylength), and the associated photoperiod response was investigated here in a mapping population developed from a cross involving a cultivar and a landrace of common bean, allowing us to identify the magnitude of QTL effects on phenotype, and their genetic and environment interactions. One important caveat in interpretation of the results is that may likely be subject to some degree of bias due to the size of the population and the marker coverage, although the study produced sufficient resolution to identify a number of QTL and some robust marker trait associations for flowering time and photoperiod response.

Time to flowering in LD and SD showed substantial variation, and the parental extremes of the photoperiod response were also observed in the RI population. We found that each parent possessed some alleles that reduce and others that increase DTF, which was reflected in transgressive segregation and indicated quantitative inheritance. The estimates of average genetic variance and broad-sense heritability for DTF were greater in LD tan in SD, and the correlation between LD and SD was positive and weak, indicating that daylength played an essential role in determining common bean flowering time in addition to daylength-independent genetic effects. The high heritability observed for DTF is consistent with previous reports in common bean (Bhakta et al., 2017) and in other species including maize (Buckler et al., 2009), tomato (Mohamed et al., 2012), and rice (Seyoum et al., 2012). Such heritability values indicate that a greater proportion of the phenotypic variation is due to genetic variation, providing opportunities for genetic improvement through selection based on the DTF trait.

The genetic complexity of flowering time and photoperiod response, and the presence of genotype by environment interaction, was supported by a total of 37 main QTL for six times to flowering and response to photoperiod traits, and more than one QTL interacting with the environment, and a high frequency of QTL epistasis in the RI population. This result provided a good opportunity for dissecting the effects of photoperiod on common bean flowering time. The number of main QTL and their associated effects varied across LD and SD, highlighting among them the QTL located on chromosomes 4 and 9. The locus on chromosome 4 was associated with both LD flowering time and photoperiod response, controlling more than 60% of phenotypic variance in some environments. The DTF-4.1 locus had a positive additive effect and showed significant QE interaction effect in LD, and opposite directions of additive × environment interaction effects in the shortest daylength environments Furthermore, the DTF-9.1, 9.4 and 9–5 QTL contributed up to 32% of the flowering time variation and were found to have main additive effects for flowering time under SD and interaction with the environment (Table 4, Figure 6, and Supplementary Tables 7, 8). Thus, our results showed that the genomic regions associated with these loci somehow control the response to different photoperiod environments and could be involved in the genetic pathway for response to photoperiod.

For each of the genomic regions mapped to chromosome 4 and 9, we identified known flowering-related genes as potential candidates. Nucleotide sequence analysis revealed the presence of polymorphisms between the parents of the mapping population for *PvCOL2* (Phvul.004G046601), *PvFD* (Phvul.009G018700), and *AGL8/FUL* (Phvul.009G203400), as well as the ortholog to the soybean maturity gene *E1* (Phvul.009G204600), which makes them promising candidate genes to underlie the QTL detected on chromosomes 4 and 9, although further studies are required for proving their role on the regulation of flowering time and photoperiod response in common bean.

## Conclusion

In conclusion, genetic analysis of time to flowering and photoperiod response in a segregating cultivated × landrace population of common bean accessions in different LD and SD environments allowed the identification of genomic regions and major QTL regulating common bean flowering. Results suggest that an approach investigating epistasis, environment, and their interactions, rather than only single QTL, is robust and effective. The two novel photoperiod sensitivity loci identified on chromosomes 4 and 9 may have played an important role in adaptation in common bean, and in future efforts should be made to identify the underlying causal molecular changes responsible for the observed flowering time phenotypic variation. Future fine-mapping and association studies that incorporate accessions from different germplasm collections will allow us to confirm the implication of the proposed candidate genes for these QTL in common bean photoperiod response.

## Data Availability Statement

The raw data supporting the conclusions of this article will be made available by the authors, without undue reservation.

## Author Contributions

MS and RL conceived the study and were in charge of overall direction and planning. AG, JV, and FY-L performed the experiments and generated the data. AG and MS wrote the manuscript, with significant contributions by JW and RL. All authors performed the experiments, analyzed the data and/or assisted in interpretation, have read and agreed to the published version of the manuscript.

## Conflict of Interest

The authors declare that the research was conducted in the absence of any commercial or financial relationships that could be construed as a potential conflict of interest.

## Footnotes

1 http://www.phytozome.net/

2 https://www.geneious.com

## Supplementary Material

Supplementary Figure 1 Profiles of climatic variables (daylength, hour; maximum and minimum temperature, °C; and solar radiation, MJ/(m^2^día)) observed during the first 100 days after sowing, at long-day (LD) and short-day (SD) environments in one location across 6 years.

Supplementary Figure 2 Distribution of days to flowering (DTF) in the RI population of a biparental cross between the cultivar Bolita (indeterminate type II, photoperiod-insensitive) and the landrace PHA1037 (indeterminate type IV, photoperiod-sensitive) grown in 12 environments; where black and white arrows correspond to Bolita and PHA1037 parents, respectively. The environments are ranging according to sowing dates from late-February to late-July (LD1-6 environments) and from mid-August to late-September (SD1-6) in each trial year (2009–2016). DTF (*X* axis) and number of RI lines (*Y* axis). The landrace PHA1037 and a proportion of RIL individuals remained vegetative until termination of the LD experiments (NF: non-flowering; 200 days).

Supplementary Figure 3 Distribution of growing degree days (GDD, °C) during each of the LD and SD environments.

Supplementary Figure 4 Distribution of the Photoperiod Response Index (PRI), response photoperiod classes 1–8 (CLASS), and the photoperiod sensitivity (PS) in the RI population of a cross between the Andean cultivar Bolita (indeterminate type II, photoperiod-insensitive) and the Andean landrace PHA1037 (indeterminate type IV, photoperiod-sensitive); where black and white arrows correspond to Bolita and PHA1037 parents, respectively. Response to photoperiod was measured as a relative change in rate of flowering under long and short day-lengths in several years. PRI values close to zero indicate non-photoperiod-sensitive flowering (stable flowering period), while values close to 30 or even higher indicate high sensitivity to photoperiod (non-flowering). PS values close to or lower than 30% could be classified as photoperiod insensitive, while values over 50% indicate high sensitivity to photoperiod. Grouping response classes 1 and 2 were classified as day-neutral, 3 and 4 as intermediate, and 5–8 as sensitive.

Supplementary Figure 5 Correlation of the genetic and physical positions and its distribution in chromosomal rearrangement regions. The blue dots represent the genetic and physical positions of markers. Total-sample data ellipses are shown as orange, solid curves. The horizontal axis represents the genetic position (cM) of the markers on the genetic linkage map. The vertical axis represents the physical position (Mb) according to the *Phaseolus vulgaris* reference genome (Phytozome). R^2^ represents the Spearman correlation coefficients between the genetic and physical positions in each chromosome.

Supplementary Table 1 Information of primer pairs and χ^2^ tests of the markers showing segregation distortion in the test cross progeny for polymorphic flowering-related gene markers.

Supplementary Table 2 DNA sequence of primer pairs used for candidate genes sequencing.

Supplementary Table 3 Genotypic data and DTF means for the 185 RILs used for the QTL analysis.

Supplementary Table 4 Estimates of means, standard errors, range of variation, variance analysis results and heritability for days to flowering (DTF) of the two common bean parents, Bolita and PHA1037, and the RI population, grown in 12 environments under Short (SD) and Long-Day (LD) conditions.

Supplementary Table 5 The main QTL detected for flowering time (DTF and GDD) and photoperiod (PRI, RRP, PS, and CLASS) traits using multiple-QTL model mapping for individual environment and year analysis.

Supplementary Table 6 Epistatic QTL detected for flowering time (DTF and GDD) and photoperiod (PRI, RRP, PS, and CLASS) traits using multiple-QTL model mapping for individual environment and year analysis.

Supplementary Table 7 The main QTL and QTL × Environment (QE) effects detected for flowering time (DTF and GDD) and photoperiod response (PRI, RRP, PS, and CLASS) traits using a multi-environment analysis.

Supplementary Table 8 Epistatic QTL and epistatic QTL × Environment (QE) effects detected for flowering time (DTF, GDD) and photoperiod (PRI, RRP, PS, and GDD) traits using multi-environment analysis.

Supplementary Table 9 Candidate genes identified in the main QTL.

Supplementary Table 10 Polymorphisms in potential candidate genes underlying flowering time QTL.
