# Supplementary material for: Characterization of QTL and Environmental Interactions Controlling Flowering Time in Andean Common Bean (Phaseolus vulgaris L.)
Source: Front Plant Sci. 2021 Jan 14;11:599462. doi: 10.3389/fpls.2020.599462 (PMC7840541; doi:10.3389/fpls.2020.599462)
Supplement: Supplementary Figure 1 — Profiles of climatic variables (daylength, hour; maximum and minimum temperature, °C; and solar radiation, MJ/(m2día)) observed during the first 100 days after sowing, at long-day (LD) and short-day (SD) environments in one location across 6 years. [file Data_Sheet_1.PDF]

**Table S1.** Information of primer pairs and χ2 tests of the markers showing segregation distortion in the test cross progeny for polymorphic flowering-related gene markers.

| **Name** | **Gene** | **Possition (phytozome)** | **Target** | **Forward primer** | **Reverse primer** | **Marker type**[a] | **Enzyme for CAPS/dCAPS** | **χ2 test for segregation distortion (skewness)**[b] |
|---|---|---|---|---|---|---|---|---|
| *PvALC* | *Phvul.001G023200* | Chr01:1931951..1935673 | *ALCATRAZ (ALC)* | CCCAGCAGGTCGGAAATAGT | CAGTCCAACGCAGCATTAAA | CAPS | *DraI* | ns |
| *PvFTc* | *Phvul.001G097200* | Chr01:18585107..18586231 | *FLOWERING LOCUS T (FT)* | AGAGGGGACTCAAAACACATATT | TTGCGGTGGAAGGAGATATT | SSRs | | ns |
| *PvFTa1* | *Phvul.001G097300* | Chr01:18602292..18604694 | *FLOWERING LOCUS T (FT)* | AATTCTCTGCTTATGGAATTGTCA | AGGCTTAAAAGAATCCACACCA | CAPS | *DraI* | ns |
| *PvFin* | *Phvul.001G189200* | Chr01:44856139..44857862 | *TERMINAL FLOWER1 (TFL1)* | CATCGAATGTGTACCTTCTCCAT | AAGGAAGCGAAACCCTAAACTATTA | dCAPS | *BccI* | ** (PHA1037) |
| *PvPHYA3* | *Phvul.001G221100* | Chr01:47642033..47647745 | *PHYTOCHROME A (PHYA)* | TGCAACACAACTTTTCAAGGA | ATCACCATCACGTCCAAACA | CAPS | *MnlI* | ** (PHA1037) |
| *PvAG2a* | *Phvul.002G243200* | Chr02:41516308..41521518 | *AGAMOUS-like 2 (AGL2)* | AAGAGTTTAAGGTTGCGAAGGA | GAAAATCAAGAACATGGTGCAA | dCAPS | *MseI* | ** (PHA1037) |
| *PvAG2b* | *Phvul.002G243200* | Chr02:41516308..41521518 | *AGAMOUS-like 2 (AGL2)* | CAGAAACTGAATAGGTGGAATTCACT | AAGAACATGGTGCAATCTCTGTATT | dCAPS | *AflI* | ** (PHA1037) |
| *PvLATE1a* | *Phvul.004G088300* | Chr04:14679773..14697707 | *GIGANTEA (GI)* | GTACGCTGGTCTATGACAAGAATA | TGTTTGCATTTCACAAACTAGAAA | dCAPS | *SspI* | ** (Bolita) |
| *PvSHP1* | *Phvul.006G034300* | Chr06:12907811..12911918 | *AGAMOUS-like 1 (AGL1)* | CCCAAGGATGTGCCTACAAG | GGTTGGGATACCCCTGAAGT | CAPS | *HpyCH4V* | ** (Bolita) |
| *PvAG1a* | *Phvul.006G169600* | Chr06:27307945..27315783 | *AGAMOUS (AG)* | TTCCCCTTTCAAAACCATTC | GAGTACATGCAGAAGAGGGTAAATA | SSRs | | ns |
| *PvAG1b* | *Phvul.006G169600* | Chr06:27307945..27315783 | *AGAMOUS (AG)* | AGGGGAAGATCAACCAAACA | GGGTTTTATTAGCAATAGCGTGAG | SSRs | | ns |
| *PvFTb3* | *Phvul.008G003700* | Chr08:337345..340888 | *FLOWERING LOCUS T-RELATED* | ACGCATGCAGAAGCAATACA | AGTTGTCGTTTGCAGGTGAAG | CAPS | *AvaII* | ns |
| *PvFDc* | *Phvul.009G018700* | Chr09:3564501..3568643 | *FLOWERING LOCUS D (FD)* | GCAAATGGATCAAGATGTTT | TAGAATAGAAGGTGAGAAGAATAATTTA | dCAPS | *DraI* | ns |
| *PvCOL3* | *Phvul.009G085300* | Chr09:13956611..13957992 | *CONSTANS-like (COL)* | CAAACAAAACAAACACAAAAACTGAT | ACCTTGGAGTCGCATGCTC | dCAPS | *BclI* | ns |
| *PvFUL1* | *Phvul.009G203400* | Chr09:30856875..30865960 | *AGAMOUS-like 8 (AGL8)* | CAATTTTTTTACTTTTTTATTTGGATATT | CCAATGGTTGAGGTACAAGGA | dCAPS | *MseI* | ns |

[a] CAPS: Cleaved Amplified Polymorphic Sequences; SSRs: Simple Sequence Repeats; dCAPS: Derived Cleaved Amplified Polymorphic Sequences.

[b] Skewed marker segregation towards Bolita or PHA1037 parental; ns,** not significant and significant at 0.01 probability level, respectively.

**Table S2.** DNA sequence of primer pairs used for candidate genes sequencing.

| Gene | Primer name | Forward primer | Reverse primer | Tª | Size band |
|---|---|---|---|---|---|
| PHYA3 | PvPHYAb-1F/1R | GCACCAGAAAGAGGGAGAGA | CCAGAAGAAGCAACAATCAGG | 58 | 366 |
| | PvPHYAb-2F/2R | GAGCATGGACACTCTGTGTGAC | ATGCTTCCCAATAGCTTGCTC | 60 | 1339 |
| | PvPHYAb-3F/R4 | TCTGGAAGCAGTGACTAGTGAGA | TCTTGTTGATGCCTAACACCAC | 60 | 1356 |
| | PvPHYAb-4F/3R | CTCCCGGAAATTGTTAGAAGG | TGCACCCTTCACTTTCTGAT | 58 | 1429 |
| ARP5 | PvRP5-1F/1R | TTGGCAAGATTGCAGAGTTAGA | CCAACATTTTCGACAAACACA | 60 | 654 |
| | PvRP5-2F/2R | GGCAGGAGAGACTCAACCAC | CCAATTTGGACAAATCAACAA | 58 | 838 |
| | PvRP5-3F/3R | TTGCTTTATGAAGACTGATGAGAT | TTGTCAAAGTTCAAAAAGAGAAAAA | 56 | 885 |
| | PvRP5-4F/4R | CCTAGCAATTCCTTGACTTGATG | TGCAACTGTTACTTGACAAAAGG | 58 | 848 |
| | PvRP5-5F/5R | TTCTCTTGTTTTTGCGGACA | TTTTTCTTTAGCCTCATTAGATTTTT | 56 | 896 |
| | PvRP5-6F/6R | TGAAGCTCGACTGTTTCAGG | AAAACTGAATAAACTGACTCGCATA | 58 | 887 |
| | PvRP5-7F/7R | CGACAACGATTGAAGCAGAA | GTGATGCCTCAGCAGATTGA | 60 | 886 |
| | PvRP5-8F/8R | TGTGCCCAAGTCAGAAACTG | TGCAATGTAAAAGAAACTCGTGA | 60 | 709 |
| RPK2 | PvRPK2-1F/1R | CATCGTTTCCATCATCGTCTT | ACAAAACCAGGCACAGAACC | 60 | 876 |
| | PvRPK2-2F/2R | ATTGGGGAGGTGCCTAGTTC | GCATATGGCGAGGAAACATT | 60 | 875 |
| | PvRPK2-3F/3R | CCCTGTATGAGCGTGTTTGA | GTACGGATTCCCAACAGCAC | 60 | 897 |
| | PvRPK2-4F/4R | CCTTCCAACAGTGGCTTGTT | GAAGTCTGGCCAATCCAAAA | 60 | 820 |
| | PvRPK2-5F/5R | ATCAGTGTGTTCCCCGTGTT | TTGAATTTAACTGGCACTCTCG | 60 | 994 |
| | PvRPK2-6F/6R | ACGCAAGACTTGGACAAAGA | TAGGCCAACCTTGTCACCAT | 58 | 994 |
| | PvRPK2-7F/7R | GGAAAACTTTGACGTAGTGATGC | GCTTAACATTTTTGCCAATC | 58 | 993 |
| | PvRPK2-8F/8R | TGAAGATTTCAGTCATTTCAAACA | TTGTGGTTCATTTGCAAAGATTA | 60 | 961 |
| | PvRPK2-9F/9R | CGCTGAATAACCAAAAGATGC | AAACTCTAATATGAAAGGAGAAATGGA | 60 | 972 |
| | PvRPK2-10F/10R | TTTTGTTGTGAAAGTGAAAAATGA | AAGCACGAAGACGAGGAAGA | 58 | 924 |
| | PvRPK2-11F/11R | AGATGTTTTCTTCTTCTGCTCATTG | CTCACCAGGAATACCCTCTTCTAGT | 60 | 908 |
| | PvRPK2-12F/12R | TGTTAATGAATTGAATGGTTCTGTG | TTGTTTTGACCAAAGTTGTGAACTA | 60 | 909 |
| | PvRPK2-13F/13R | GTTCAGGAGAATTCACTTTTCACAG | TATTTCAATAGAGCTCAAGCCATTC | 60 | 879 |
| | PvRPK2-14F/14R | GCTCAATAACAACAATCTTTCTGGT | TCTTGTGAAGAATCCTCCAATCTAC | 60 | 779 |
| | PvRPK2-15F/15R | AATCTTGAAAAGTTCATCCAGGAG | CAGAACAGCACAAAAGATATTGAAA | 60 | 897 |
| COL2 | PvCOL-1F/1R | TCTCCCCCAACACTACTTGG | GAATCTGTGTTCCGGTTCGT | 60 | 563 |
| | PvCOL-2F/2R | CTCATCTTGGGTCCCCTCTT | TTTTCATGACCAAACCTAAACTTG | 60 | 667 |
| | PvCOL-3F/3R | TGCTGTCGCAATCTTTATGG | GGGTGAAAAGAAGCAGAAAAA | 58 | 695 |
| | PvCOL-4F/4R | TTCTCTTTTGCATCTCCTCTTTTT | GTGCCTTCCAATGATGTGGT | 60 | 698 |
| AP3 | PvAP3-1F/1R | CCCCACCTTCCCTTTCTATTT | TGTATATAGGTAATTGGGGCATATAAA | 60 | 899 |
| | PvAP3-2F/2R | TTGTTCGTGAGCGTAAGGTG | GGTAGATTTCAAAAATTAATGAGCAA | 58 | 827 |
| | PvAP3-3F/3R | TATCCTTGGTGGGAATGGAG | TCAAACAATTTCTTGTGTGTCCTT | 60 | 879 |
| | PvAP3-4F/4R | TCCTTTCAACAAATCTTCTCAGG | TCCAAGCTCAAACCAAAGTT | 58 | 900 |
| | PvAP3-5F/5R | TTGATCTTGATCTTCTAATGTCCA | TCAATGGGAGATGATAGGTTAATG | 58 | 895 |
| FDc | PvFDc-1F/1R | GTGTCAATGTCCGTGTGCAT | CTCCGTCAAACGAGCAATTT | 60 | 874 |
| | PvFDc-2F/2R | CTCTGAACATGTCTGGAACGA | AAAATCGATGCCAATCTGTG | 58 | 701 |
| | PvFDc-3F/3R | AGCCCTTTGAGTAATGTAATCTCC | GCGATTACATGGAAAAGTGTGA | 58 | 871 |
| | PvFDc-4F/4R | CTTTTCCATGTAATCGCTCCTT | TGAACCTACAGTGGAACAACTAGG | 60 | 790 |
| | PvFDc-5F/5R | CAACGTTTTCCTTCAATTCG | GGCGTAATCAGACCGTAATG | 58 | 720 |
| | PvFDc-6F/6R | TGCATGTAGGTGTCCCAAAC | TTTTCCCCTTTCCATCCTCT | 60 | 756 |
| | PvFDc-7F/7R | TGGAGTAATCCGATAGCACACA | TTAGGTTGGAGGAAGGAAGTAGC | 60 | 890 |
| E1 | PvE1-1F/1R | AACCTTGGCTGCGAGTTTAC | TGTTTGTGATTCCCTCAACG | 58 | 643 |
| | PvE1-2F/2R | CACTTCCATTTCACAAATTCCA | AAGGATGTGGATGTGAAGCAC | 60 | 832 |
| FUL1 | PvFUL1-1F/1R | GCCAATGATATTTGCTCTTTTAATTT | GCGACTTCAGCATCACAAAG | 60 | 537 |
| | PvFUL1-2F/2R | GGTGCAGTTGAAGAGGATCG | AGGGTTGAAGGTACGTGAGG | 58 | 204 |
| | PvFUL1-3F/3R | GCATGAAGCTAACTTGTATGGTGT | AGGGATCAAAATTGAAGATTGG | 58 | 360 |
| | PvFUL1-4F/4R | TTGCCCTTCTACACAGATCATT | TTGAAACTATATCAGGAAAATGCAC | 58 | 356 |
| | PvFUL1-5F/5R | GCAGTAGTAGTGCATTTTCCTGA | CCACCCTTTGTTGATTCGTT | 58 | 214 |
| | PvFUL1-6F/6R | CACATGGATGGAGTTAACTTTGTT | GCTTGTTGTGCTGCTAGTGC | 60 | 207 |
| | PvFUL1-7F/7R | AAAAACCAGATAAAGGAAAAAGAGA | TCAACGTCAATTAAATGAATGGTA | 58 | 215 |
| | PvFUL1-8F/8R | TGAGTAGGGTTATTAAAACATTGTGC | GGCCATATTCATATTTAATTTTTATGC | 60 | 214 |

**Table S4**. Estimates of means, standard errors, range of variation, variance analysis results and heritability for days to flowering (DTF) of the two common bean parents, Bolita and PHA1037, and the RI population, grown in twelve environments under Short (SD) and Long-Day (LD) conditions.

| Environment | Block effect | Parents | | | RILs | | | |
|---|---|---|---|---|---|---|---|---|
| | | PMB0225 | PHA-1037 | $P_{PAR}$ | Mean | Range | $P_{RIL}$ | $h^2$ |
| LD1 | | 46.5 ± 1.50 | 200.0 ± 0.00 | *** | 67.9 ± 3.48 | 35 - 200 | ** | 0.98 ± 0.001 |
| LD2 | | 46.5 ± 1.50 | 200.0 ± 0.00 | *** | 53.1 ± 0.96 | 41 - 200 | ** | 0.96 ± 0.006 |
| LD3 | | 55.7 ± 1.20 | 200.0 ± 0.00 | *** | 83.4 ± 1.76 | 53 - 200 | *** | 0.98 ± 0.002 |
| LD4 | | 51.5 ± 0.50 | 200.0 ± 0.00 | *** | 75.9 ± 2.02 | 43 - 200 | *** | 0.98 ± 0.003 |
| LD5 | *** | 49.0 ± 1.15 | 200.0 ± 0.00 | *** | 79.9 ± 2.29 | 40 - 200 | *** | 0.98 ± 0.002 |
| LD6 | | 41.0 ± 0.15 | 91.5 ± 1.50 | ** | 52.9 ± 0.52 | 37 - 85 | *** | 0.85 ± 0.022 |
| SD1 | *** | 48.0 ± 0.30 | 64.0 ± 0.00 | ** | 60.3 ± 0.30 | 44 - 78 | *** | 0.48 ± 0.059 |
| SD2 | *** | 46.0 ± 0.40 | 66.0 ± 0.02 | ** | 51.5 ± 0.22 | 41 - 61 | *** | 0.43 ± 0.057 |
| SD3 | * | 44.0 ± 0.02 | 61.0 ± 0.58 | *** | 50.4 ± 0.23 | 34 - 62 | *** | 0.68 ± 0.068 |
| SD4 | | 46.3 ± 0.33 | 64.7 ± 0.33 | * | 50.3 ± 0.19 | 41 - 65 | *** | 0.86 ± 0.017 |
| SD5 | | 49.0 ± 0.71 | 57.5 ± 0.50 | * | 51.0 ± 0.17 | 42 - 65 | *** | 0.47 ± 0.06 |
| SD6 | | 47.5 ± 2.50 | 68.0 ± 3.00 | * | 56.8 ± 0.41 | 45 - 83 | *** | 0.74 ± 0.028 |

*, **, *** significant at the 0.05, 0.01 and 0.001 probability levels, respectively, for difference among parents ($P_{PAR}$; $t_{test}$), RILs ($P_{RIL}$), and block effect, $h^2$, heritability estimates and their s.e.

**Table S5**. The main QTL detected for flowering time (DTF and GDD) and photoperiod (PRI, RRP, PS and CLASS) traits using multiple-QTL model mapping for individual environment and year analysis.

| Env. | QTL | Position in cM[a] | Position in Mb[a] | Chromosome | Marker interval | F (F threshold) | $R^{2c}$ | A[d] | |
|---|---|---|---|---|---|---|---|---|---|
| **Days to flowering (DTF)** | | | | | | | | | |
| SD1 | DTF-4.1 | 24.02-25.09 | 4.11-5.86 | 4 | BMc155-Pv04G048200 | 23.52 (12.05) | 8.77 | 3.05 | *** |
| | DTF-9.5 | 62.16-63.66 | 30.86-30.84 | 9 | PvFUL1-Pv09G203300 | 16.52 (12.05) | 9.20 | 1.43 | *** |
| SD2 | DTF-1.3 | 80.86-90.91 | 44.85-47.64 | 1 | PvFin-PvPHYA3 | 15.52 (9.89) | 6.99 | 2.49 | *** |
| | DTF-7.2 | 83.24-95.79 | 50.69 | 7 | E31M61-110-BMc216 | 14.96 (9.89) | 7.90 | -1.68 | *** |
| SD3 | DTF-1.4 | 90.91-102.59 | 47.64-49.04 | 1 | PvPHYA3-BMc324 | 14.77 (12.32) | 10.25 | 3.93 | *** |
| | DTF-9.4 | 57.57-57.57 | 30.99-31.06 | 9 | Pv09G204500-Pv09G204700 | 18.31 (12.32) | 13.88 | 4.81 | *** |
| SD4 | DTF-4.1 | 24.02-25.09 | 4.11-5.86 | 4 | BMc155-Pv04G048200 | 12.52 (11.51) | 1.67 | 1.30 | ** |
| | DTF-5.2 | 47.13-49.57 | 33.90 | 5 | E32M60-263-PvCh05-33.9 | 13.85 (11.51) | 4.65 | 0.82 | ** |
| | DTF-9.4 | 57.57-57.57 | 30.99-31.06 | 9 | Pv09G204500-Pv09G204700 | 11.58 (11.51) | 32.14 | 2.63 | *** |
| | DTF-10.1 | 0.00-12.24 | | 10 | E31M31-173-E31M50-168 | 22.33 (11.51) | 5.04 | 0.65 | * |
| SD5 | Non significant | Non significant | Non significant | | | | | | |
| SD6 | DTF-9.1 | 10.15-13.24 | 1.86-3.56 | 9 | BM154-PvFDc | 28.05 (11.95) | 16.43 | 3.10 | *** |
| | DTF-9.5 | 62.16-63.66 | 30.86-30.84 | 9 | PvFUL1-Pv09G203300 | 20.55 (11.95) | 14.10 | 2.73 | *** |
| LD1 | DTF-4.1 | 24.02-25.09 | 4.11-5.86 | 4 | BMc155-Pv04G048200 | 28.12 (10.38) | 21.78 | 30.19 | *** |
| LD2 | DTF-4.1 | 24.02-25.09 | 4.11-5.86 | 4 | BMc155-Pv04G048200 | 35.08 (10.52) | 19.26 | 11.76 | ** |
| LD3 | Non significant | Non significant | Non significant | | | | | | |
| LD4 | DTF-4.1 | 24.02-25.09 | 4.11-5.86 | 4 | BMc155-Pv04G048200 | 30.80 (11.43) | 55.25 | 60.19 | *** |
| LD5 | DTF-4.1 | 24.02-25.09 | 4.11-5.86 | 4 | BMc155-Pv04G048200 | 74.71 (12.61) | 62.42 | 77.45 | *** |
| | DTF-9.5 | 62.16-63.66 | 30.86-30.84 | 9 | PvFUL1-Pv09G203300 | 20.68 (12.61) | 1.80 | 9.14 | *** |
| LD6 | DTF-1.4 | 90.91-102.59 | 47.64-49.04 | 1 | PvPHYA3-BMc324 | 20.90 (12.12) | 5.11 | 12.65 | *** |
| | DTF-4.1 | 24.02-25.09 | 4.11-5.86 | 4 | BMc155-Pv04G048200 | 24.46 (12.12) | 30.28 | 10.46 | *** |
| **Growing degree days (GDD)** | | | | | | | | | |
| SD1 | GDD-4.1 | 24.02-25.09 | 4.11-5.86 | 4 | BMc155-Pv04G048200 | 23.45 (13.72) | 8.8 | 26.25 | *** |
| | GDD-9.4 | 57.57-57.57 | 30.99-31.06 | 9 | Pv09G204500-Pv09G204700 | 16.61 (13.72) | 9.06 | 13.72 | *** |
| SD2 | GDD-1.4 | 90.91-102.59 | 47.64-49.04 | 1 | PvPHYA3-BMc324 | 12.69 (12.01) | 10.18 | 39.03 | *** |
| | GDD-7.2 | 83.24-95.79 | 50.69 | 7 | E31M61-110-BMc216 | 14.06 (12.01) | 7.37 | -12.22 | *** |
| SD3 | GDD-1.4 | 90.91-102.59 | 47.64-49.04 | 1 | PvPHYA3-BMc324 | 16.54 (12.72) | 11.47 | 46.55 | *** |
| | GDD-9.4 | 57.57-57.57 | 30.99-31.06 | 9 | Pv09G204500-Pv09G204700 | 14.90 (12.74) | 12.38 | 26.41 | *** |
| | GDD-7.1 | 45.52-52.59 | 28.75-31.12 | 7 | P-BMc294 | 16.68 (12 .74) | 2.38 | -8.26 | *** |
| SD4 | GDD-4.1 | 24.02-25.09 | 4.11-5.86 | 4 | BMc155-Pv04G048200 | 12.42 (11.79) | 6.67 | 7.81 | *** |
| | GDD-5.2 | 47.13-49.57 | 33.90 | 5 | E32M60-263-PvCh05-33.9 | 13.60 (11.79) | 4.59 | 5.82 | *** |
| | GDD-9.4 | 57.57-57.57 | 30.99-31.06 | 9 | Pv09G204500-Pv09G204700 | 11.88 (11.79) | 32.23 | 19.26 | *** |
| | GDD-8.1 | 23.75-28.09 | 7.06-7.85 | 8 | BMc121-BM165 | 13.28 (11.79) | 5.05 | 6.79 | *** |
| | GDD-10.1 | 0.00-12.24 | | 10 | E31M31-173-E31M50-168 | 22.96 (8.79) | 5.25 | 4.08 | * |
| SD5 | GDD-4.6 | 41.01-41.65 | 10.00-26.75 | 4 | Pv04G94900-Chr04.10.01 | 15.57 (11.70) | 7.27 | -3.94 | *** |
| SD6 | GDD-9.1 | 10.15-13.24 | 1.86-3.56 | 9 | BM154-PvFDc | 32.87 (12.88) | 17.53 | 7.81 | *** |
| LD1 | GDD-4.1 | 24.02-25.09 | 4.11-5.86 | 4 | BMc155-Pv04G048200 | 25.81 (10.84) | 20.36 | 205.9 | *** |
| LD2 | GDD-4.1 | 24.02-25.09 | 4.11-5.86 | 4 | BMc155-Pv04G048200 | 33.38 (10.35) | 18.4 | 90.12 | *** |
| LD3 | GDD-4.1 | 24.02-25.09 | 4.11-5.86 | 4 | BMc155-Pv04G048200 | 13.39 (12.98) | 30.75 | 209.5 | *** |
| LD4 | GDD-4.1 | 24.02-25.09 | 4.11-5.86 | 4 | BMc155-Pv04G048200 | 123.73 (11.83) | 53.87 | 467.87 | *** |
| LD5 | GDD-4.1 | 24.02-25.09 | 4.11-5.86 | 4 | BMc155-Pv04G048200 | 78.35 (12.72) | 64.03 | 675.33 | *** |
| | GDD-9.4 | 57.57-57.57 | 30.99-31.06 | 9 | Pv09G204500-Pv09G204700 | 27.18 (12.72) | 1.81 | 92.82 | *** |
| LD6 | GDD-1.4 | 90.91-102.59 | 47.64-49.04 | 1 | PvPHYA3-BMc324 | 23.40 (13.24) | 5.72 | 133.28 | *** |
| | GDD-4.1 | 24.02-25.09 | 4.11-5.86 | 4 | BMc155-Pv04G048200 | 25.99 (13.24) | 28.83 | 103.71 | *** |

## **Table S5**. Continued.

| Env. | QTL | Position in cM[a] | Position in Mb[a] | Chromosome | Marker interval | F (F threshold) | $R^2$[c] | A[d] | |
|---|---|---|---|---|---|---|---|---|---|
| **Photoperiod Response Index (PRI)** | | | | | | | | | |
| 2009 | PRI-4.1 | 24.02-25.09 | 4.11-5.86 | 4 | BMc155-Pv04G048200 | 13.47 (12.15) | 19.83 | 26.12 | *** |
| 2010 | PRI-1.1 | 14.34-30.73 | 18.60 | 1 | E32M60-147-PvFTa1 | 14.50 (11.16) | 8.41 | 6.89 | *** |
| | PRI-4.1 | 24.02-25.09 | 4.11-5.86 | 4 | BMc155-Pv04G048200 | 22.88 (11.16) | 51.31 | 54.35 | *** |
| 2011 | PRI-4.1 | 24.02-25.09 | 4.11-5.86 | 4 | BMc155-Pv04G048200 | 27.64 (9.69) | 16.10 | 10.13 | *** |
| 2013 | PRI-4.1 | 24.02-25.09 | 4.11-5.86 | 4 | BMc155-Pv04G048200 | 14.04 (11.23) | 20.09 | 10.70 | *** |
| 2015 | PRI-4.1 | 24.02-25.09 | 4.11-5.86 | 4 | BMc155-Pv04G048200 | 283.65 (11.76) | 61.98 | 78.10 | *** |
| | PRI-9.5 | 62.16-63.66 | 30.86-30.84 | 9 | PvFUL1-Pv09G203300 | 12.91 (11.76) | 1.87 | 7.36 | ** |
| 2016 | PRI-4.1 | 24.02-25.09 | 4.11-5.65 | 4 | BMc155-Pv04G048200 | 66.42 812.98) | 39.5 | 13.59 | *** |
| | PRI-5.1 | 41.86-43.50 | 30.40-11.24 | 5 | PvCh05-30.4-BMc321 | 13.63 (12.98) | 4.03 | 12.65 | *** |
| **Percentage of photoperiod sensitivity (PS)** | | | | | | | | | |
| 2009 | Non significant | Non significant | Non significant | | | | | | |
| 2010 | PS-4.1 | 24.02-25.09 | 4.11-5.86 | 4 | BMc155-Pv04G048200 | 97.63 (13.55) | 47.93 | 28.23 | *** |
| | PS-9.2 | 25.05-29.34 | 13.96-15.10 | 9 | PvCOL3-PvCh09-15.1 | 14.87 (13.55) | 9.32 | -5.94 | *** |
| 2011 | Non significant | Non significant | Non significant | | | | | | |
| 2013 | PS-4.1 | 62.16-63.66 | 4.11-5.86 | 4 | BMc155-Pv04G048200 | 43.50 (10.97) | 20.97 | 3.9 | *** |
| | PS-9.5 | 24.02-25.09 | 30.86-30.84 | 9 | PvFUL1-Pv09G203300 | 16.59 (10.97) | 4.92 | 1.77 | *** |
| 2015 | PS-4.1 | 62.16-63.66 | 4.11-5.86 | 4 | BMc155-Pv04G048200 | 84.92 (12.12) | 63.07 | 39.67 | *** |
| | PS-9.4 | 57.57-57.57 | 30.99-31.06 | 9 | Pv09G204500-Pv09G204700 | 28.71 (12.12) | 4.87 | 3.85 | *** |
| | PS-9.5 | 62.16-63.66 | 30.86-30.84 | 9 | PvFUL1-Pv09G203300 | 31.97 (12.12) | 2.17 | 8.44 | *** |
| 2016 | PS-4.1 | 24.02-25.09 | 4.11-5.86 | 4 | BMc155-Pv04G048200 | 63.18 (12.50) | 39.24 | 14.79 | *** |
| | PS-5.1 | 41.86-43.50 | 30.40-11.24 | 5 | PvCh05-30.4-BMc321 | 14.06 (12.50) | 4.14 | 4.74 | *** |
| **Relative Response to Photoperiod (RRP)** | | | | | | | | | |
| 2009 | Non significant | Non significant | Non significant | | | | | | |
| 2010 | RRP-4.1 | 24.02-25.09 | 4.11-5.86 | 4 | BMc155-Pv04G048200 | 21.36 (11.50) | 46.96 | 0.25 | *** |
| 2011 | RRP-4.1 | 24.02-25.09 | 4.11-5.86 | 4 | BMc155-Pv04G048200 | 42.13 (12.07) | 22.81 | 0.10 | *** |
| 2013 | RRP-4.1 | 24.02-25.09 | 4.11-5.86 | 4 | BMc155-Pv04G048200 | 46.16 (11.18) | 21.97 | 0.06 | *** |
| | RRP-9.5 | 62.16-63.66 | 30.86-30.84 | 9 | PvFUL1-Pv09G203300 | 17.26 (11.18) | 4.95 | 0.03 | *** |
| 2015 | RRP-4.1 | 24.02-25.09 | 4.11-5.86 | 4 | BMc155-Pv04G048200 | 79.15 (12.06) | 61.44 | 0.49 | *** |
| | RRP-9.5 | 62.16-63.66 | 30.86-30.84 | 9 | PvFUL1-Pv09G203300 | 35.04 (12.06) | 3.27 | 0.01 | *** |
| 2016 | RRP-4.1 | 24.02-25.09 | 4.11-5.86 | 4 | BMc155-Pv04G048200 | 64.36 (12.38) | 38.66 | 0.23 | *** |
| | RRP-5.1 | 41.86-43.50 | 30.40-11.24 | 5 | PvCh05-30.4-BMc321 | 12.49 (12.28) | 3.71 | 0.09 | *** |
| **Photoperiod response on a scale of 1-8 (CLASS)** | | | | | | | | | |
| 2009 | Non significant | Non significant | Non significant | | | | | | |
| 2010 | CLASS-4.1 | 24.02-25.09 | 4.11-5.86 | 4 | BMc155-Pv04G048200 | 87.37 (11.81) | 45.19 | 2.82 | *** |
| 2013 | CLASS-4.4 | 34.37-34.58 | 18.37-18.68 | 4 | Pv04G091700-Pv04G92400 | 18.88 (10.48) | 17.38 | -0.63 | *** |
| 2013 | CLASS-4.5 | 35.76-36.04 | 14.42-16.56 | 4 | Chr04.14.42-Chr04.16.56 | 18.14 (10.48) | 13.34 | 0.14 | *** |
| | CLASS-7.2 | 83.24-95.79 | 50.69 | 7 | E31M61-110-BMc216 | 10.79 (10.48) | 6.55 | -0.14 | *** |
| 2015 | CLASS-4.1 | 24.02-25.09 | 4.11-5.86 | 4 | BMc155-Pv04G048200 | 81.30 (12.45) | 62.16 | 3.94 | *** |
| | CLASS-9.5 | 62.16-63.66 | 30.86-30.84 | 9 | PvFUL1-Pv09G203300 | 37.52 (12.45) | 2.90 | 1.09 | *** |
| 2016 | CLASS-4.1 | 24.02-25.09 | 4.11-5.86 | 4 | BMc155-Pv04G048200 | 60.34 (12.57) | 38.20 | 1.51 | *** |
| | CLASS-5.1 | 41.86-43.50 | 30.40-11.24 | 5 | PvCh05-30.4-BMc321 | 16.55 (12.57) | 4.90 | 0.56 | *** |

[a] Estimated confidence interval of QTL position in cM (Kosambi, 1943) and Mb (Phytozome web).
[b] Chr = chromosome.
[c] F values of significance of each QTL. The critical F-value was determined by a permutation test of 1000 repetitions at the confidence level of 95% (Churchill and Doerge, 1994).
[d] Percentage of the phenotypic variation explained by additive effects.
[e] Estimated additive effect. Positive values indicate that alleles from PHA1037 increase the trait value, and negative values indicate that positive effect is due to the presence of the alleles from Bolita. Experiment-wide P value. *P≤0.05, **P≤0.01, ***P≤0.001.
Trial years for photoperiod response traits: 2009 (LD1 *vs.* SD2), 2010 (LD4 *vs.* SD6), 2011 (LD2 *vs.* SD3), 2013 (LD6 *vs.* SD1), 2015 (LD5 *vs.* SD4), and 2016 (LD3 *vs.* SD5).

**Table S6**. Epistatic QTL detected for flowering time (DTF and GDD) and photoperiod (PRI, RRP, PS and CLASS) traits using multiple-QTL model mapping for individual environment and year analysis.

| Env. | QTL (position)[a] | Chr[b] | Marker interval | QTL (position)[a] | Chr[b] | Marker interval | F (F threshold)[c] | $R^2$[d] | Aa[e] |
|---|---|---|---|---|---|---|---|---|---|
| **Days to flowering (DTF)** | | | | | | | | | |
| SD1 | DTF-5.1 (41.86-43.50) (30.40-11.24) | 5 | PvCh05-30.4-BMc321 | DTF-8.1 (23.75-28.09) (41.52-41.52) | 8 | BMc121-BM165 | 11.77 (8.91) | 9.68 | 4.28 *** |
| SD5 | DTF-7.2 (83.24-95.79) (50.69) | 7 | E31M61-110-BMc216 | DTF-2.3 (65.21-65.77) (41.52-41.52) | 2 | PvAG2b-PvAG2a | 18.11 (7.27) | 9.61 | -3.26 *** |
| LD3 | DTF-4.1 (24.02-25.09) (4.11-5.86) | 4 | BMc155-Pv04G048200 | DTF-1.4 (90.91-102.59) (47.64-49.04) | 1 | PvPHYA3-BMc324 | 24.12 (10.19) | 9.34 | 26.54 *** |
| | DTF-4.1 (24.02-25.09) (4.11-5.86) | 4 | BMc155-Pv04G048200 | DTF-3.2 (89.47-95.46) (47.39) | 3 | E45M50-389-BM189 | 36.83 (10.19) | 14.51 | 44.29 *** |
| | DTF-4.1 (24.02-25.09) (4.11-5.86) | 4 | BMc155-Pv04G048200 | DTF-5.1 (41.86-43.50) (30.40-11.24) | 5 | PvCh05-30.4-BMc321 | 23.14 (10.19) | 6.68 | 5.59 *** |
| LD4 | DTF-4.3 (32.65-33.02) (33.50-41.88) | 4 | BM140-Pv04G103000 | DTF-9.3 (29.33-33.71) (15.10-16.74) | 9 | PvCh09-15.1-PV-at007 | 38.2 (11.59) | 9.9 | -37.88 *** |
| **Growing degree days (GDD)** | | | | | | | | | |
| LD3 | GDD-4.2 (31.61-32.23) (12.73-11.16) | 4 | BM171-IAC91 | GDD-5.1 (41.86-43.50) (41.86-43.50) | 5 | PvCh05-30.4-BMc321 | 25.15 (10.85) | 7.53 | 233.12 *** |
| LD4 | GDD-4.3 (32.64-33.02) (33.50-41.88) | 4 | BM140-Pv04G103000 | GDD-9.3 (29.33-33.71) (15.10-16.74) | 9 | PvCh09-15.1-PV-at007 | 35.97 (11.87) | 10.80 | -320.85 *** |
| LD5 | GDD-4.1 (24.02-25.09) (4.11-5.86) | 4 | BMc155-Pv04G048200 | GDD-9.4 (57.57-57.57)(30.99-31.04) | 9 | Pv09G204500-Pv09G204 | 8.96 (8.00) | 1.21 | 101.32 *** |
| | GDD-9.4 (57.57-57.57) (30.99-31.06) | 9 | Pv09G204500-Pv09G204 | GDD-10.2 (44.73-45.85) (3.50-6.93) | 10 | IAC061-BMc234 | 8.14 (8.00) | 1.47 | -56.14 *** |
| **Photoperiod Response Index (PRI)** | | | | | | | | | |
| 2010 | PRI-4.1 (24.02-25.09) (4.11-5.86) | 4 | BMc155-Pv04G048200 | PRI-1.1(14.34-30.73)(18.60) | 1 | E32M60-147-PvFTa1 | 13.67 (12.41) | 5.62 | 19.54 *** |
| | PRI-4.2 (31.61-32.23) (12.73-11.16) | 4 | BM171-IAC91 | PRI-8.1 (23.75-28.09) (7.06-7.85) | 8 | BMc121-BM165 | 20.25 (12.41) | 12.07 | 48.28 *** |
| 2013 | PRI-4.3 (32.64-33.02) (33.50-41.88) | 4 | BM140-Pv04G103000 | PRI-5.1 (41.86-43.50) (30.40-11.24) | 5 | PvCh05-30.4-BMc321 | 18.43 (11.56) | 1.51 | 0.47 ** |
| 2015 | PRI-4.1 (24.02-25.09) (4.11-5.86) | 4 | BMc155-Pv04G048200 | PRI-9.5 (62.16-63.66) (30.86-30.84) | 9 | PvFUL1-Pv09G203300 | 12.36 (11.98) | 1.50 | 12.45 *** |
| 2016 | PRI-8.3 (45.36-45.58) (48.34) | 8 | BMc316-E45M50-69 | PRI-6.2 (12.72-13.47) | 6 | E45M50-167-E40M60-91 | 18.50 (11.79) | 9.29 | 14.88 *** |
| **Percentage of photoperiod sensitivity (PS)** | | | | | | | | | |
| 2010 | PS-2.2 (15.58-16.80) (26.8-27.03) | 2 | Chr02.26.8-PvM115 | PS-4.3 (32.64-33.02) (33.50-41.88) | 4 | BM140-Pv04G103000 | 13.25 (10.83) | 6.95 | -10.85 *** |
| | PS-4.1 (24.02-25.09) (4.11-5.86) | 4 | BMc155-Pv04G048200 | PS-9.2 (25.05-29.34)(13.96-15.10) | 9 | PvCOL3-PvCh09-15.1 | 12.98 (10.83) | 5.55 | -8.77 *** |
| 2011 | PS-2.1 (14.23-14.44) (48.51) | 2 | E31M50-104-Chr02.48.5 | PS-4.2 (31.61-32.23) (12.73-11.16) | 4 | BM171-IAC91 | 22.60 (8.30) | 12.00 | 8.49 *** |
| | PS-2.2 (15.58-16.80) (26.8-27.03) | 2 | Chr02.26.8-PvM115 | PS-4.2 (31.61-32.23) (12.73-11.16) | 4 | BM171-IAC91 | 12.63 (8.30) | 7.03 | -4.52 *** |
| 2015 | PS-9.4 (57.57-57.57) (30.99-31.06) | 9 | Pv09G204500-Pv09G204 | PS-10.1 (0.00-12.24) | 10 | E31M31-173-E31M50-16 | 13.75 (10.0) | 1.80 | -3.01 *** |
| 2016 | PS-8.3 (45.36-45.58) (48.34) | 8 | BMc316-E45M50-69 | PS-6.1 (7.26-7.497) | 6 | E45M38-113-E45M38-11 | 22.89 (11.27) | 6.89 | 6.50 *** |
| | PS-9.2 (25.05-29.34)(13.96-15.10) | 9 | PvCOL3-PvCh09-15.1 | PS-2.4 (65.67-67.12) (41.52-42.12) | 2 | PvAG2a-Chr02.42.12 | 17.31 (11.27) | 5.41 | 3.35 *** |

**Table S6**. Continued.

| Env. | QTL (position)[a] | Chr[b] | Marker interval | QTL (position)[a] | Chr[b] | Marker interval | F (F threshold)[c] | $R^{2d}$ | Aa[e] |
|---|---|---|---|---|---|---|---|---|---|
| **Relative Response to Photoperiod (RRP)** | | | | | | | | | |
| 2010 | RRP-2.1 (14.23-14.44) (48.51) | 2 | E31M50-104-Chr02.48.5 | RRP-4.2 (31.61-32.23) (12.73-11.16) | 4 | BM171-IAC91 | 14.29 (10.12) | 3.02 | 0.02 * |
| | RRP-2.2 (15.58-16.80) (26.8-27.03) | 2 | Chr02.26.8-PvM115 | RRP-4.2 (31.61-32.23) (12.73-11.16) | 4 | BM171-IAC91 | 10.54 (10.12) | 6.81 | -0.18 *** |
| | RRP-4.2 (31.61-32.23) (12.73-11.16) | 4 | BM171-IAC91 | RRP-8.1 (23.75-28.09) (7.06-7.85) | 8 | BMc121-BM165 | 51.94 (10.12) | 3.58 | 0.19 *** |
| 2011 | RRP-2.1 (14.23-14.44) (48.51) | 2 | E31M50-104-Chr02.48.5 | RRP-4.2 (31.61-32.23) (12.73-11.16) | 4 | BM171-IAC91 | 17.54 (9.80) | 6.58 | 0.06 *** |
| | RRP-2.2 (15.58-16.80) (26.8-27.03) | 2 | Chr02.26.8-PvM115 | RRP-4.2 (31.61-32.23) (12.73-11.16) | 4 | BM171-IAC91 | 19.35 (9.80) | 8.02 | -0.07 *** |
| 2013 | RRP-4.1 (24.02-25.09) (4.11-5.86) | 4 | BMc155-Pv04G048200 | RRP-9.5 (62.16-63.66) (30.86-30.84) | 9 | PvFUL1-Pv09G203300 | 23.04 (13.45) | 21.74 | 0.09 *** |
| 2015 | RRP-9.4 (57.57-57.57) (30.99-31.06) | 9 | Pv09G204500-Pv09G204 | RRP-8.2 (37.81-39.54) (14.67-19.06) | 8 | PVEST194-IAC027 | 10.98 (9.06) | 2.01 | -0.08 *** |
| **Photoperiod response on a scale of 1-8 (CLASS)** | | | | | | | | | |
| 2010 | CLASS-2.2 (15.58-16.80) (26.8-27.03) | 2 | Chr02.26.8-PvM115 | CLASS-4.3 (32.64-33.02) (33.50-41.88) | 4 | BM140-Pv04G103000 | 31.21 (12.08) | 9.60 | -1.30 *** |
| 2011 | CLASS-2.1 (14.23-14.44) (48.51) | 2 | E31M50-104-Chr02.48.5 | CLASS-4.2 (31.61-32.23) (12.73-11.16) | 4 | BM171-IAC91 | 23.80 (7.97) | 12.53 | 0.80 *** |
| | CLASS-2.2 (15.58-16.80) (26.8-27.03) | 2 | Chr02.26.8-PvM115 | CLASS-4.2 (31.61-32.23) (12.73-11.16) | 4 | BM171-IAC91 | 11.38 (7.97) | 6.38 | -0.37 *** |
| 2013 | CLASS-4.1 (24.02-25.09) (4.11-5.86) | 4 | BMc155-Pv04G048200 | CLASS-9.5 (62.16-63.66) (30.86-30.84) | 9 | PvFUL1-Pv09G203300 | 39.80 (8.47) | 13.24 | 0.28 *** |
| 2015 | CLASS-9.4 (57.57-57.57) (30.99-31.04) | 9 | Pv09G204500-Pv09G204 | CLASS-8.2 (37.81-39.54) (14.67-19.06) | 8 | PVEST194-IAC027 | 12.47 (9.76) | 1.91 | -0.57 *** |
| 2016 | CLASS-3.1 (41.40-44.79) (28.219-32.13) | 3 | BMd1-PVEST042 | CLASS-9.6 (67.57-68.57) | 9 | E40M50-47-E40M50-51 | 31.21 (12.08) | 9.60 | -1.30 *** |
| | CLASS-5.1 (41.86-43.50) (30.40-11.24) | 5 | PvCh05-30.4-BMc321 | CLASS-1.2 (32.54-35.08) (18.59-30.54) | 1 | PvFTc-BM200 | 12.61 (12.08) | 2.85 | -0.26 *** |

[a] Estimated confidence interval of QTL position in brackets (in cM, Kosambi, 1943; and Mb, Phytozome web).

[b] Chr. = chromosome.

[c] F values of significance of each QTL. The critical F-value was determined by a permutation test of 1000 repetitions at the confidence level of 95% (Churchill and Doerge, 1994).

[d] Percentage of the phenotypic variation explained by additive by additive epistatic effects.

[e] Estimated additive by additive epistatic effect. Positive values indicate that alleles from PHA1037 increase the trait value, and negative values indicate that positive effect is due to the presence of the alleles from Bolita. Experiment-wide P value. *P≤0.05, **P≤0.01, ***P≤0.001.

Trial years for photoperiod response traits: 2009 (LD1 *vs.* SD2), 2010 (LD4 *vs* SD6), 2011 (LD2 *vs.* SD3), 2013 (LD6 *vs.* SD1), 2015 (LD5 *vs.* SD4), and 2016 (LD3 *vs.* SD5).

**Table S7.** The main QTL and QTL x Environment (QE) effects detected for flowering time (DTF and GDD) and photoperiod response (PRI, RRP, PS and CLASS) traits using a multi-environment analysis.

| QTL | Marker interval | Position[a] | Chr[b] | F (F threshold)[c] | A[d] | *P* | $R^2$(a)[e] | QE AE[f] | | | | | | $R^2$ (ae)[g] | $R^2$(ae)[h] | | | | | |
|---|---|---|---|---|---|---|---|---|---|---|---|---|---|---|---|---|---|---|---|---|
| | | | | | | | | LD1 | LD2 | LD3 | LD4 | LD5 | LD6 | | LD1 | LD2 | LD3 | LD4 | LD5 | LD6 |
| **Days to flowering (DTF)** | | | | | | | | | | | | | | | | | | | | |
| DTF-4.1 | BMc155-Pv04G048200 | 24.02-25.09 | 4 | 31.81 (4.31) | 34.22 | *** | 31.94 | -8.45 * | -20.18 *** | | 16.97 *** | 37.94 *** | -24.99 *** | 14.48 | 2.40 | 2.84 | | 1.50 | 8.99 | 4.19 |
| DTF-9.4 | Pv09G204500-Pv09G204700 | 57.57-57.57 | 9 | 7.67 (4.31) | 5.15 | *** | 0.88 | | | | | | | 0.17 | | | | | | |
| **Growing degree days (GDD)** | | | | | | | | | | | | | | | | | | | | |
| GDD-4.1 | BMc155-Pv04G048200 | 24.02-25.09 | 4 | 32.26 (4.28) | 275.59 | *** | 32.49 | -104.39 *** | -169.34 *** | | 119.72 *** | 331.83 *** | -187.18 *** | 15.37 | 0.57 | 3.07 | | 1.23 | 1.04 | 3.60 |
| GDD-9.4 | Pv09G204500-Pv09G204700 | 57.57-60.47 | 9 | 9.51 (4.28) | 52.72 | *** | 0.57 | | | | | 29.45 * | | 0.29 | | | | | 0.06 | 0.07 |
| **QTL** | **Marker interval** | **Position[a]** | **Chr[b]** | **F (F threshold)[c]** | **A[d]** | ***P*** | **$R^2$(a)[e]** | **QE AE[f]** | | | | | | **$R^2$ (ae)[g]** | **$R^2$(ae)[h]** | | | | | |
| | | | | | | | | SD1 | SD2 | SD3 | SD4 | SD5 | SD6 | | SD1 | SD2 | SD3 | SD4 | SD5 | SD6 |
| **Days to flowering (DTF)** | | | | | | | | | | | | | | | | | | | | |
| DTF-9.1 | BM154-PvFDc | 10.15-13.24 | 9 | 12.69 (4.32) | 1.22 | *** | 5.19 | | | | | -0.71 * | 2.18 *** | 4.25 | | | | | 0.51 | 4.15 |
| DTF-9.4 | Pv09G204500-Pv09G204700 | 57.57-57.57 | 9 | 13.37 (4.32) | 3.55 | *** | 9.80 | | | | | | -0.99 ** | 6.18 | | | | | | 2.04 |
| **Growing degree days (GDD)** | | | | | | | | | | | | | | | | | | | | |
| GDD-1.4 | PvPHYA3-BMc324 | 90.91-102.59 | 1 | 9.34 (4.18) | 34.29 | *** | 7.44 | | | 17.93 * | | -16.35 * | -17.98 * | 1.35 | | | 0.61 | | 0.40 | 0.50 |
| GDD-9.4 | Pv09G204500-Pv09G204700 | 57.57-57.57 | 9 | 6.76 (4.18) | 5.99 | *** | 3.14 | | | | | | | 0.47 | | | | | | |
| **QTL** | **Marker interval** | **Position[a]** | **Chr[b]** | **F (F threshold)[c]** | **A[d]** | ***P*** | **$R^2$(a)[e]** | **QE AE[f]** | | | | | | **$R^2$ (ae)[g]** | **$R^2$(ae)[h]** | | | | | |
| | | | | | | | | 2009 | 2010 | 2011 | 2013 | 2015 | 2016 | | 2009 | 2010 | 2011 | 2013 | 2015 | 2016 |
| **Photoperiod Response Index (PRI)** | | | | | | | | | | | | | | | | | | | | |
| PRI-1.5 | BMc324-E36M31-121 | 102.59-113.51 | 1 | 5.30 (4.27) | -2.50 | *** | 1.91 | | -3.82 * | | | | | 2.06 | | 0.76 | | | | |
| PRI-4.1 | BMc155-Pv04G048200 | 24.02-25.09 | 4 | 30.87 (4.27) | 33.77 | *** | 25.71 | -25.68 *** | 27.56 *** | -19.15 *** | -31.29 *** | 45.79 *** | | 18.42 | 2.13 | 2.5 | 2.17 | 5.38 | 10.11 | |
| PRI-9.2 | PvCOL3-PvCh09-15.1 | 25.05-29.34 | 9 | 4.39 (4.27) | -0.41 | ns | 0.13 | -7.68 *** | | | | 5.93 *** | 5.04 *** | 1.33 | 0.37 | | | 0.16 | 0-32 | |
| PRI-9.4 | Pv09G204500-Pv09G204700 | 57.57-57.57 | 9 | 4.79 (4.27) | 2.99 | *** | 0.10 | | | | | 4.69 * | | 0.32 | | | | 0.02 | | |
| **Percentage of photoperiod sensitivity (PS)** | | | | | | | | | | | | | | | | | | | | |
| PS-4.1 | BMc155-Pv04G048200 | 24.02-25.09 | 4 | 26.66 (4.19) | 17.39 | *** | 28.26 | -15.77 *** | 10.08 *** | -5.26 *** | -13.91 *** | 23.92 *** | | 15.51 | 2.46 | 1.45 | 0.73 | 4.27 | 9.97 | |
| PS-9.2 | PvCOL3-PvCh09-15.1 | 25.05-29.34 | 9 | 6.01 (4.19) | -0.62 | ns | 0.16 | -4.03 ** | -4.89 *** | 2.28 * | | 3.43 *** | 1.99 * | 2.01 | 0.38 | 1.24 | 0.03 | | 0.28 | 0.33 |
| PS-9.4 | Pv09G204500-Pv09G204700 | 57.57-60.47 | 9 | 7.33 (4.19) | -2.31 | *** | 0.68 | | | -2.53 * | | | | 1.60 | | | 0.07 | | | |
| PS-9.5 | PvFUL1-Pv09G203300 | 62.16-63.66 | 9 | 7.83 (4.19 | 2.41 | *** | 0.44 | | | | | 3.63 *** | | 1.07 | | | | | 0.71 | |
| **Relative Response to Photoperiod (RRP)** | | | | | | | | | | | | | | | | | | | | |
| RRP-1.4 | PvPHYA3-BMc324 | 90.91-102.59 | 1 | 4.88 (4.10) | -0.01 | * | 1.74 | | -0.02 * | | | 0.02 * | | 1.65 | | 0.80 | | | 0.09 | |
| RRP-4.1 | BMc155-Pv04G048200 | 24.02-25.09 | 4 | 25.96 (4.10) | 0.19 | *** | 26.95 | -0.22 *** | 0.15 *** | -0.07 *** | -0.15 *** | 0.26 *** | | 14.03 | 2.79 | 1.55 | 0.83 | 3.87 | 7.84 | |
| RRP-9.2 | PvCOL3-PvCh09-15.1 | 25.05-29.34 | 9 | 6.32 (4.10) | -0.02 | *** | 1.28 | -0.08 *** | -0.04 ** | | | 0.05 *** | 0.04 *** | 1.49 | 0.37 | 0.84 | | | 0.15 | |
| RRP-9.5 | PvFUL1-Pv09G203300 | 62.16-63.66 | 9 | 7.80 (4.10) | 0.01 | * | 1.66 | -4.00 *** | | | | 0.05 *** | | 1.40 | 0.82 | | | | 0.81 | |
| **Photoperiod response on a scale of 1-8 (CLASS)** | | | | | | | | | | | | | | | | | | | | |
| CLASS-1.6 | PVEST076-E32M51-329 | 114.10-115.60 | 1 | 5.23 (4.16) | -0.08 | * | 1.3 | | -0.19 * | | | | | 1.35 | | 0.79 | | | | |
| CLASS-4.1 | BMc155-Pv04G048200 | 24.02-25.09 | 4 | 27.03 (4.16) | 1.49 | *** | 27.55 | -1.49 *** | 01.09 *** | -0.49 *** | -1.23 *** | 2.10 *** | | 15.14 | 2.1 | 1.68 | 0.76 | 4.37 | 9.53 | |
| CLASS-9.2 | PvCOL3-PvCh09-15.1 | 25.05-29.34 | 9 | 6.19 (4.16) | -9.00 | * | 0.16 | -0.54 *** | -0.34 *** | | | | 0.30 ** | 1.83 | 0.72 | 0.72 | | | | |
| CLASS-9.5 | PvFUL1-Pv09G203300 | 62.16-63.66 | 9 | 5.96 (4.16) | 0.18 | *** | 0.3 | -0.54 *** | | | | 0.71 *** | | 1.23 | 0.66 | | | | 0.67 | |

**Table S7.** Continued.

[a] Estimated confidence interval of QTL position (in Kosambi cM).
[b] Chromosome (Chr).
[c] F values of significance of each QTL. The critical F-value was determined by a permutation test of 1000 repetitions at the confidence level of 95% (Churchill and Doerge, 1994).
[d] Predicted additive effect. Positive values indicate that alleles from PHA1037 increase the trait value, and negative values indicate that positive effect on the traits is due to the presence of the alleles from Bolita.
[e] Percentage of the phenotypic variation explained by additive effects.
[f] Predicted additive by environment interaction effect. The meaning of sign values is described in the footnote [d].
[g] Percentage of the phenotypic variation explained by additive x environment interaction effect.
[h] Percentage of the phenotypic variation explained by additive x environment interaction effect in each environment and year.
*P ≤ 0.05, **P ≤ 0.01, ***P ≤ 0.001. Only significant effects are listed.

**Table S8**. Epistatic QTL and epistatic QTL x Environment (QE) effects detected for flowering time (DTF, GDD) and photoperiod (PRI, RRP, PS and GDD) traits using multi-environment analysis.

| QTLi[a] | Marker interval | Chr (position)[b] | QTLj[a] | Marker interval | Chr (position)[b] | F (F threslod)[c] | Aa[d] | $R^2$ (aa)[e] | epistatic QE AAE[f] | | | | | | $R^2$(aae)[g] | $R^2$ (aae)[g] | | | | | |
|---|---|---|---|---|---|---|---|---|---|---|---|---|---|---|---|---|---|---|---|---|---|
| | | | | | | | | | LD1 | LD2 | LD3 | LD4 | LD5 | LD6 | | LD1 | LD2 | LD3 | LD4 | LD5 | LD6 |
| DTF-4.1 | BMc155-Pv04G048200 | 4 (24.02-25.09) | DTF-9.4 | Pv09G204500-Pv09G204700 | 9 (57.57-57.57) | 8.15 (4.26) | 8.99 *** | 2.20 | | | 9.55 *** | | | -5.88 * | 0.96 | | | 0.60 | | | 0.33 |
| DTF-1.6 | E36M31-83-PVEST076 | 1 (113.62-114.10) | DTF-4.2 | BM171-IAC91 | 4 (31.61-32.23) | 7.35 (4.26) | 10.09 *** | 2.17 | | | 6.79 * | | | | 0.97 | | | 0.43 | | | |
| DTF-4.3 | BM140-Pv04G103000 | 4 (32.65-33.02) | DTF-9.7 | BMc184-PvCOL3 | 9 (20.25-25.05) | 5.30 (4.26) | -3.09 * | 0.05 | -11.47 * | | 13.46 *** | -20.21 *** | 12.89 *** | | 2.74 | 0.56 | | 0.58 | 1.50 | 0.51 | |
| GDD-1.9 | BM53-IAC93 | 1 (41.14-42.15) | GDD-4.1 | BMc155-Pv04G048200 | 4 (24.02-25.09) | 5.19 (4.29) | 3.71 ns | 0.00 | | -51.02 * | | | | | 0.93 | | 0.49 | | | | |
| GDD-1.6 | E36M31-83-PVEST076 | 1 (113.62-114.10) | GDD-4.2 | BM171-IAC91 | 4 (31.61-32.23) | 10.53 (4.29) | 81.77 *** | 2.51 | | | 47.97 * | | | -42.76 * | 0.98 | | | 0.38 | | | 0.29 |
| GDD-4.3 | BM140-Pv04G103000 | 4 (32.65-33.02) | GDD-9.7 | BMc184-PvCOL3 | 9 (20.25-25.05) | 6.18 (4.29) | -20.97 ns | 0.00 | -90.98 * | | 101.49 *** | -169.09 *** | 111.35 *** | | 2.76 | 0.52 | | 0.49 | 1.51 | 0.63 | |
| **QTLi[a]** | **Marker interval** | **Chr (position)[b]** | **QTLj[a]** | **Marker interval** | **Chr (position)[b]** | **F (F threslod)[c]** | **Aa[d]** | **$R^2$ (aa)[e]** | **epistatic QE AAE[f]** | | | | | | **$R^2$(aae)[g]** | **$R^2$ (aae)[g]** | | | | | |
| | | | | | | | | | **SD1** | **SD2** | **SD3** | **SD4** | **SD5** | **SD6** | | **SD1** | **SD2** | **SD3** | **SD4** | **SD5** | **SD6** |
| DTF-1.7 | BM200-E43M38-138 | 1 (35.08-37.36) | DTF-2.5 | GATS91-BMc367 | 2 (0.0-8.139 | 4.50 (3.97) | -0.34 * | 0.70 | | | | | | -1.73 *** | 2.97 | | | | | | 2.76 |
| DTF-1.8 | PvCh01-10.6-BM53 | 1 (40.27-41.14) | DTF-2.6 | Chr02.42.26-Chr02.42.23 | 2 (62.98-63.26) | 5.59 (3.97) | -0.88 *** | 1.74 | | | | | | | 1.29 | | | | | | |
| GDD-2.6 | Chr02.42.26-Chr02.42.2 | 2 (62.98-63.26) | GDD-7.2 | E31M61-110-BMc216 | 7 (83.24-95.79) | 4.66 (4.07) | -12.51 *** | 3.47 | -15.33 *** | | | | | | 1.14 | 0.91 | | | | | |
| GDD-4.1 | BMc155-Pv04G048200 | 4 (24.02-25.09) | GDD-7.1 | P-BMc294 | 7 (45.52-52.59) | 4.16 (4.07) | 7.60 *** | 0.46 | 10.43 *** | | | | -8.48 * | -7.40 * | 1.58 | 0.73 | | | | 0.51 | |
| GDD-5.3 | IAC286-PvCh05-30.4 | 5 (35.93-41.86) | GDD-8.5 | E31M50-580-BMc330 | 8 (41.36-42.09) | 4.35 (4.07) | 10.40 *** | 1.76 | | | | | | | 1.10 | | | | | | |
| **QTLi[a]** | **Marker interval** | **Chr (position)[b]** | **QTLj[a]** | **Marker interval** | **Chr (position)[b]** | **F (F threslod)[c]** | **Aa[d]** | **$R^2$ (aa)[e]** | **epistatic QE AAE[f]** | | | | | | **$R^2$(aae)[g]** | **$R^2$ (aae)[g]** | | | | | |
| | | | | | | | | | **2009** | **2010** | **2011** | **2013** | **2015** | **2016** | | **2009** | **2010** | **2011** | **2013** | **2015** | **2016** |
| PRI-6.3 | PvCh06-19.5-E45M50-50 | 6 (10.90-11.43) | PRI-8.4 | BM211-PVEST194 | 8 (37.67.37.81) | 6.21 (4.29) | 6.54 *** | 1.79 | 11.28 * | | | | -14.22 *** | 12.15 ** | 1.88 | 0.90 | | | | 0.10 | 0.54 |
| PRI-6.3 | PvCh06-19.5-E45M50-50 | 6 (10.90-11.43) | PRI-8.2 | PVEST194-IAC027 | 8 (37.81-39.54) | 4.86 (4.29) | 3.93 * | 0.94 | 11.59 ** | | | | | | 1.71 | 1.20 | | | | | |
| PS-4.7 | SNP-4564-BSNP-49 | 4 (0.0-10.40) | PS-9.2 | PvCOL3-PvCh09-15.1 | 9 (25.05-29.34) | 5.64 (4.06) | 0.52 ns | 0.90 | | -7.15 *** | | | | 5.14 *** | 2.03 | | | | | | 0.64 |
| RRP-1.6 | E36M31-83-PVEST076 | 1 (113.62-114.10) | RRP-4.2 | BM171-IAC91 | 4 (31.61-32.23) | 7.00 (4.32) | 0.06 *** | 1.84 | | | | -0.03 * | | | 0.65 | | | | 0.26 | | |
| RRP-9.2 | PvCOL3-PvCh09-15.1 | 9 (25.05-29.34) | RRP-9.5 | PvFUL1-Pv09G203300 | 9 (62.16-63.66) | 4.44 (4.32) | -0.03 *** | 1.57 | | | | | -3.00 * | | 0.55 | | | | | 0.33 | |
| CLASS-1.10 | PVEST270-IAC21 | 1 (72.68-74.509 | CLASS-9.8 | E31M51-59-Pv09G227300 | 9 (70.27-73.68) | 6.49 (4.34) | -0.28 *** | 1.17 | | | | -0.33 *** | | | 1.24 | | | | 0.57 | | |
| CLASS-1.6 | E36M31-83-PVEST076 | 1 (113.62-114.10) | CLASS-4.2 | BM171-IAC91 | 4 (31.61-32.23) | 9.29 (4.34) | 0.48 *** | 2.21 | | | | | | | 0.72 | | | | | | |
| CLASS-4.1 | BMc155-Pv04G048200 | 4 (24.02-25.09) | CLASS-9.5 | PvFUL1-Pv09G203300 | 9 (62.16-63.66) | 5.10 (4.34) | 0.34 * | 0.68 | | | 0.79 *** | | | | 0.43 | | | 0.38 | | | |
| CLASS-4.1 | BMc155-Pv04G048200 | 4 (24.02-25.09) | CLASS-9.2 | PvCOL3-PvCh09-15.1 | 9 (25.05-29.34) | 4.33 (4.34) | 0.21 * | 0.35 | | -0.97 *** | | | 0.41 * | 0.38 * | 1.63 | | 1.22 | | | 0.32 | 0.28 |

[a] QTLi and QTLj are the pair of QTLs involved in epistatic interaction.
[b] Chromosome (Chr.) and the estimated confidence interval of QTL position in brackets (in Kosambi cM).
[c] F values of significance of each QTL. The critical F-value was determined by a permutation test of 1000 repetitions at the confidence level of 95% (Churchill and Doerge 1994).
[d] Estimated additive by additive epistatic effect. Positive values indicate that alleles from PHA1037 increase the trait value, and negative values indicate that positive effect on the traits is due to the presence of the alleles from Bolita.
[e] Percentage of the phenotypic variation explained by additive by additive epistatic effects.
[f] Predicted additive by additive epistatic effect by environment interaction effect. Epistasis associated with environments. The meaning of sign values is described in the footnote [d].
[g] Percentage of the phenotypic variation explained by additive by additive epistatic effect by environment interaction effect.
*P ≤ 0.05, **P ≤ 0.01, ***P ≤ 0.001. Only significant effects are listed. ns = No significant effects on the five environmental conditions evaluated.

**Table S10.** Polymorphisms in potential candidate genes underlying flowering time QTL.

| QTL | Gene | | Polymorphism genome location | SNP | | | InDel | | | Polymorphism gene position | Functional consequence |
|---|---|---|---|---|---|---|---|---|---|---|---|
| | | | | Reference genome (*P. vulgaris* V2.1) | PHA1037 | BOLITA | Reference | PHA1037 | BOLITA | | |
| DTF1..4 | *PvPHYA3* | Phvul.001G221100 | Chr01:47,647,439 | G | G | A | - | - | - | CDS (5th exon) | Non-synonymous substitution (Gly-1066-Ser) |
| DTF1..4 | *PvARP5* | Phvul.001G232900 | Chr01:48,672,298 | A | C | C | - | - | - | CDS (12th exon) | Non-synonymous substitution (Glu-631-Ala) |
| | | | Chr01:48,672,828 | A | A | T | - | - | - | CDS (11th exon) | Non-synonymous substitution (Glu-494-Val) |
| DTF-4.1 | *PvRPK2* | Phvul.004G037600 | Chr04:4,360,349 | A | G | G | - | - | - | 2nd intron | - |
| | | | Chr04:4,361,034 | A | G | G | - | - | - | 2nd intron | - |
| | | | Chr04:4,362,329 | C | T | T | - | - | - | 2nd intron | - |
| | | | Chr04:4,367,511 | C | G | G | - | - | - | 2nd intron | - |
| DTF-4.1 | *PvCOL2* | Phvul.004G046601 | Chr04:5,650,186 | A | T | A | - | - | - | 5'UTR (1st exon) | - |
| DTF-9.1 | *PvAP3* | Phvul.009G013900 | - | - | - | - | - | - | - | - | - |
| DTF-9.1 | *PvFDc* | Phvul.009G018700 | Chr09:3,565,457 | T | T | G | - | - | - | 2nd intron | - |
| | | | Chr09:3,566,057 | G | G | C | - | - | - | 2nd intron | - |
| | | | Chr09:3,567,128 | C | T | T | - | - | - | 2nd intron | - |
| DTF-9.4 | *PvE1* | Phvul.009G204600 | Chr09:31,042,488 | - | - | - | AATTAATC GTTGAGG GAATCACA AACATAAC CTCTATA | AATTAATC GTTGAGG GAATCACA AACATAAC CTCTATA | GTCT | 3' region (at 29 bp downstream of the stop codon) | - |
| DTF-9.5 | *PvFUL1* | Phvul.009G203400 | Chr09:30,864,591 | - | - | - | AA | AAA | AA | 6th intron | - |

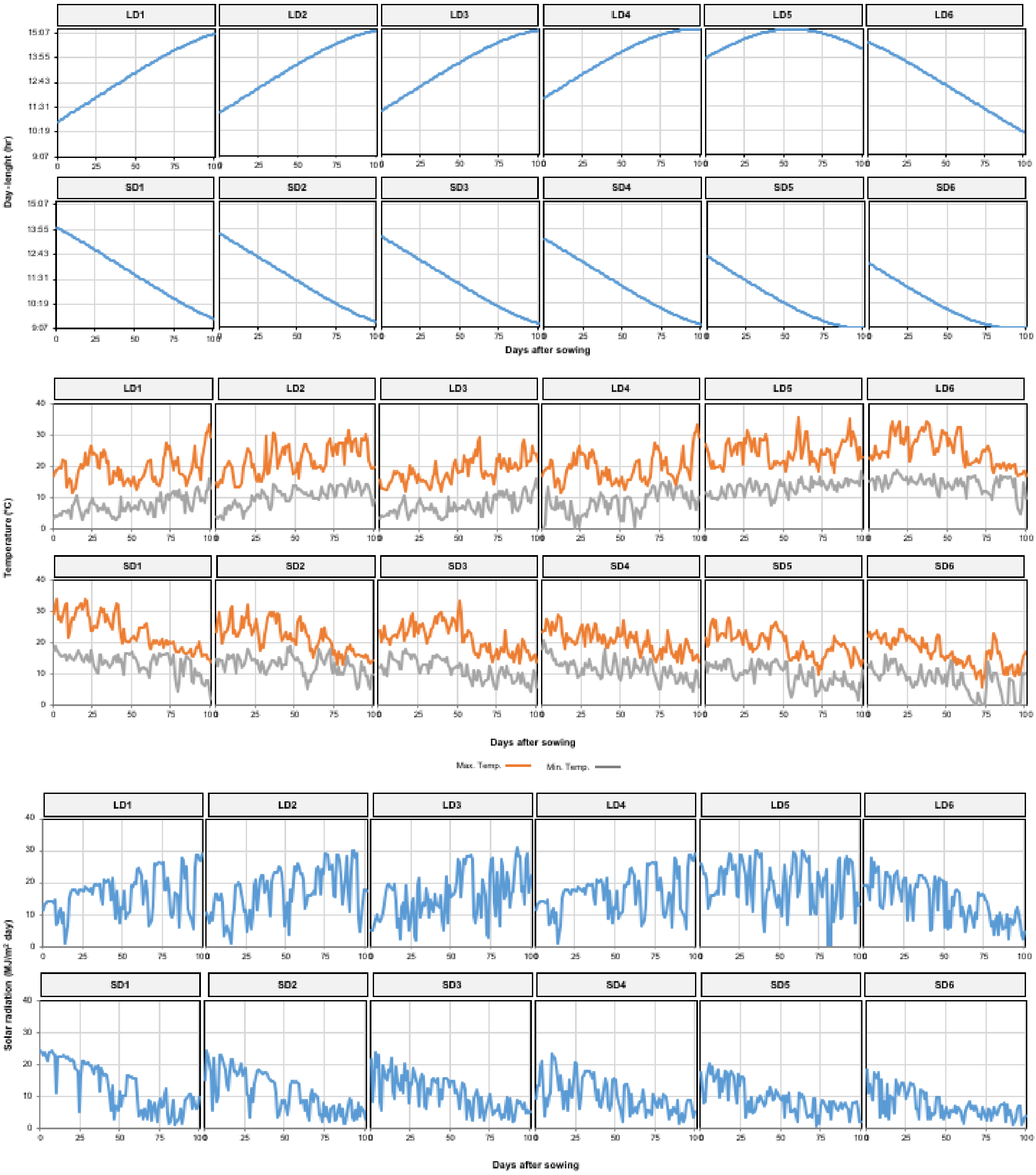


**Fig. S1.** Profiles of climatic variables (daylength, hr; maximum and minimum temperature, °C; and solar radiation, MJ / (m$^2$día)) observed during the first 100 days after sowing, at long-day (LD) and short-day (SD) environments in one location across six years.

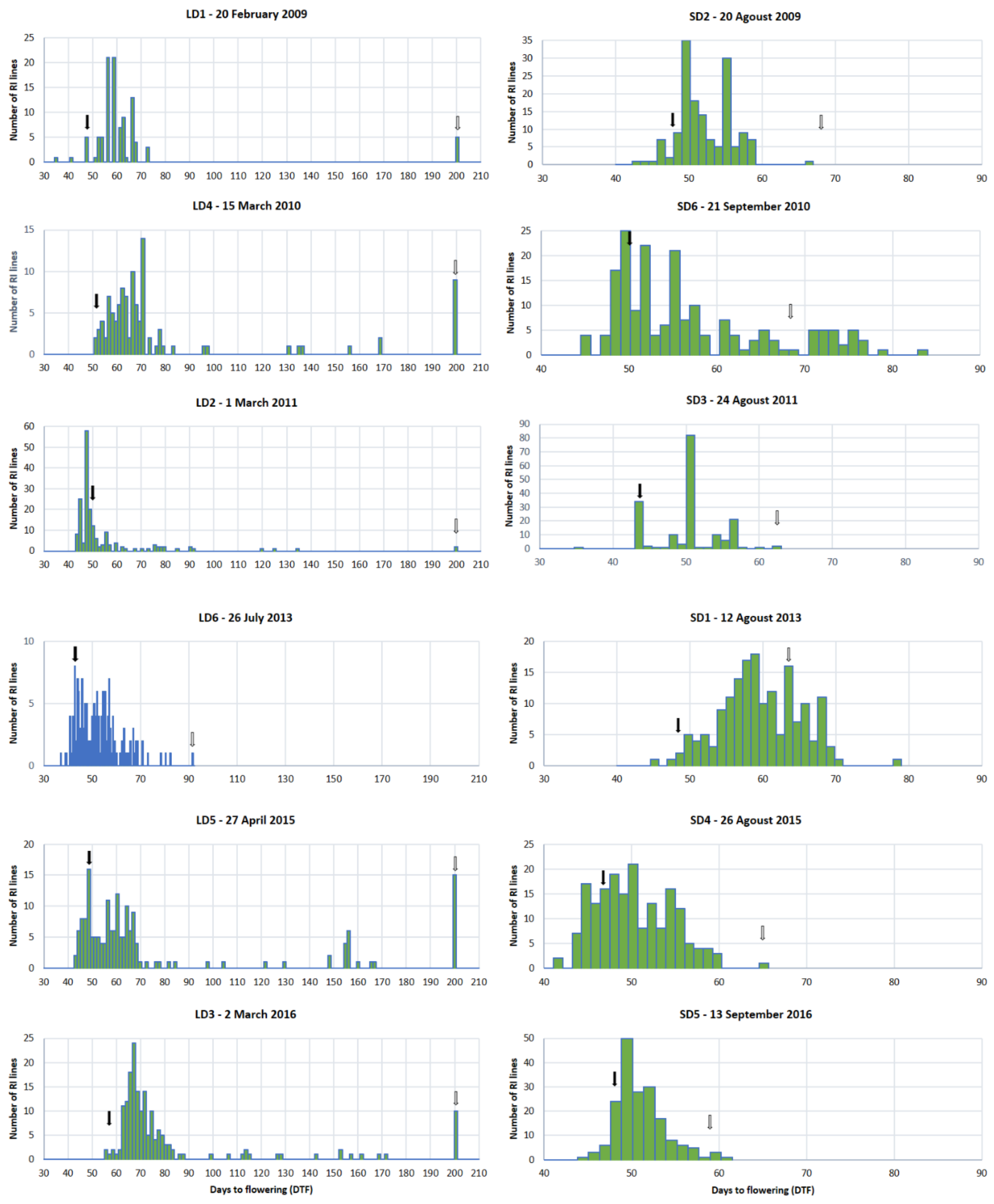


**Fig. S2.** Distribution of days to flowering (DTF) in the RI population of a biparental cross between the cultivar Bolita (indeterminate type II, photoperiod-insensitive) and the landrace PHA1037 (indeterminate type IV, photoperiod-sensitive) grown in twelve environments; where black and white arrows correspond to Bolita and PHA1037 parents, respectively. The environments are ranging according to sowing dates from late-February to late-July (LD1-6 environments) and from mid-August to late-September (SD1-6) in each trial year (2009-2016). DTF (X axis) and number of RI lines (Y axis). The landrace PHA1037 and a proportion of RIL individuals remained vegetative until termination of the LD experiments (NF: non-flowering; 200 days).

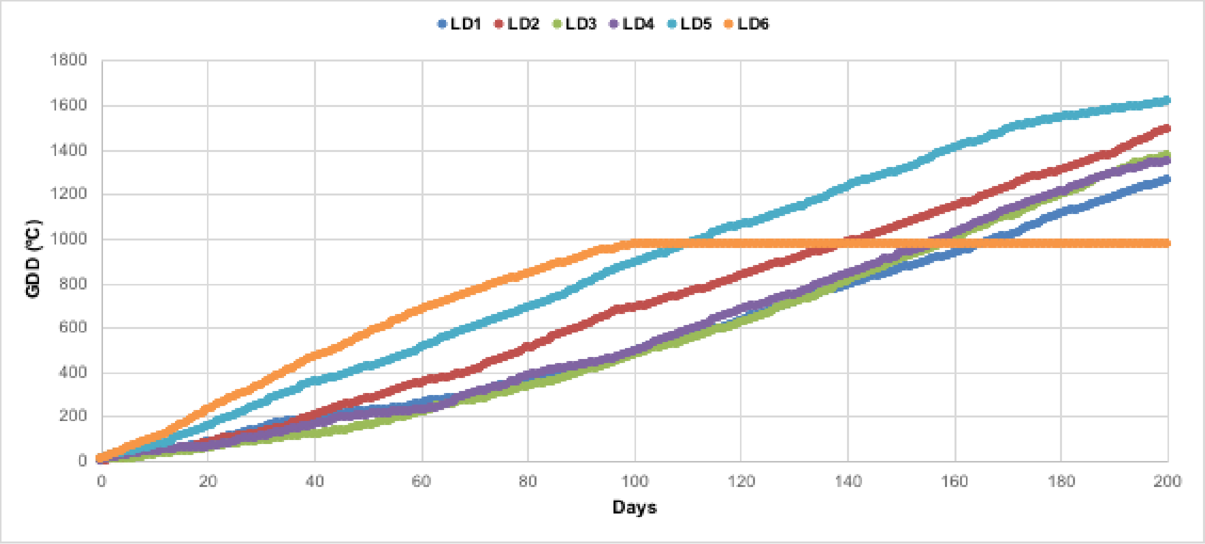


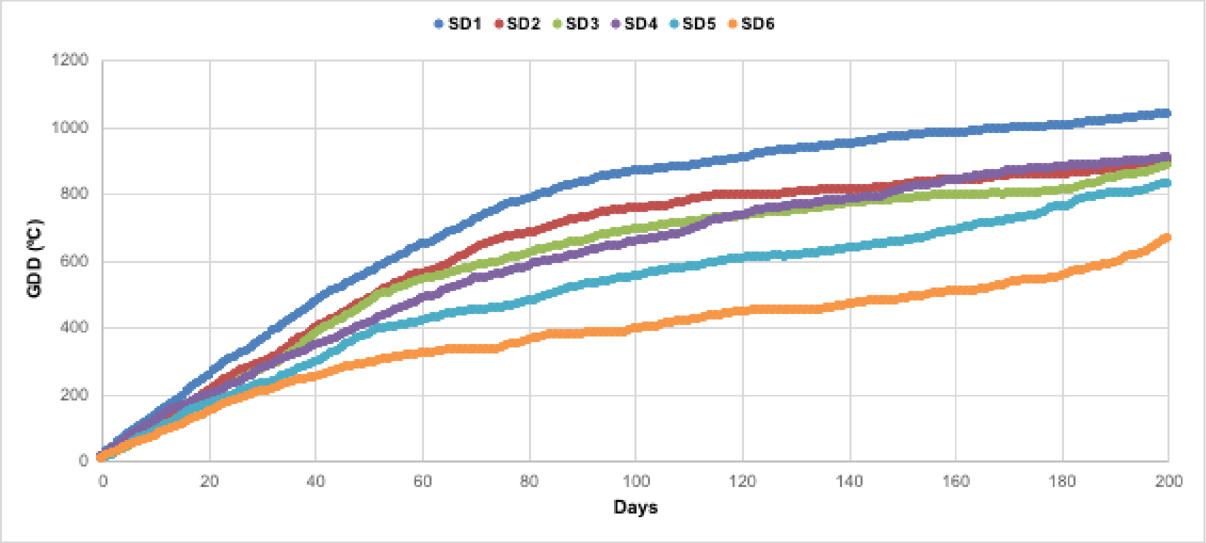


**Fig. S3.** Distribution of growing degree days (GDD, °C) during each of the LD and SD environments.

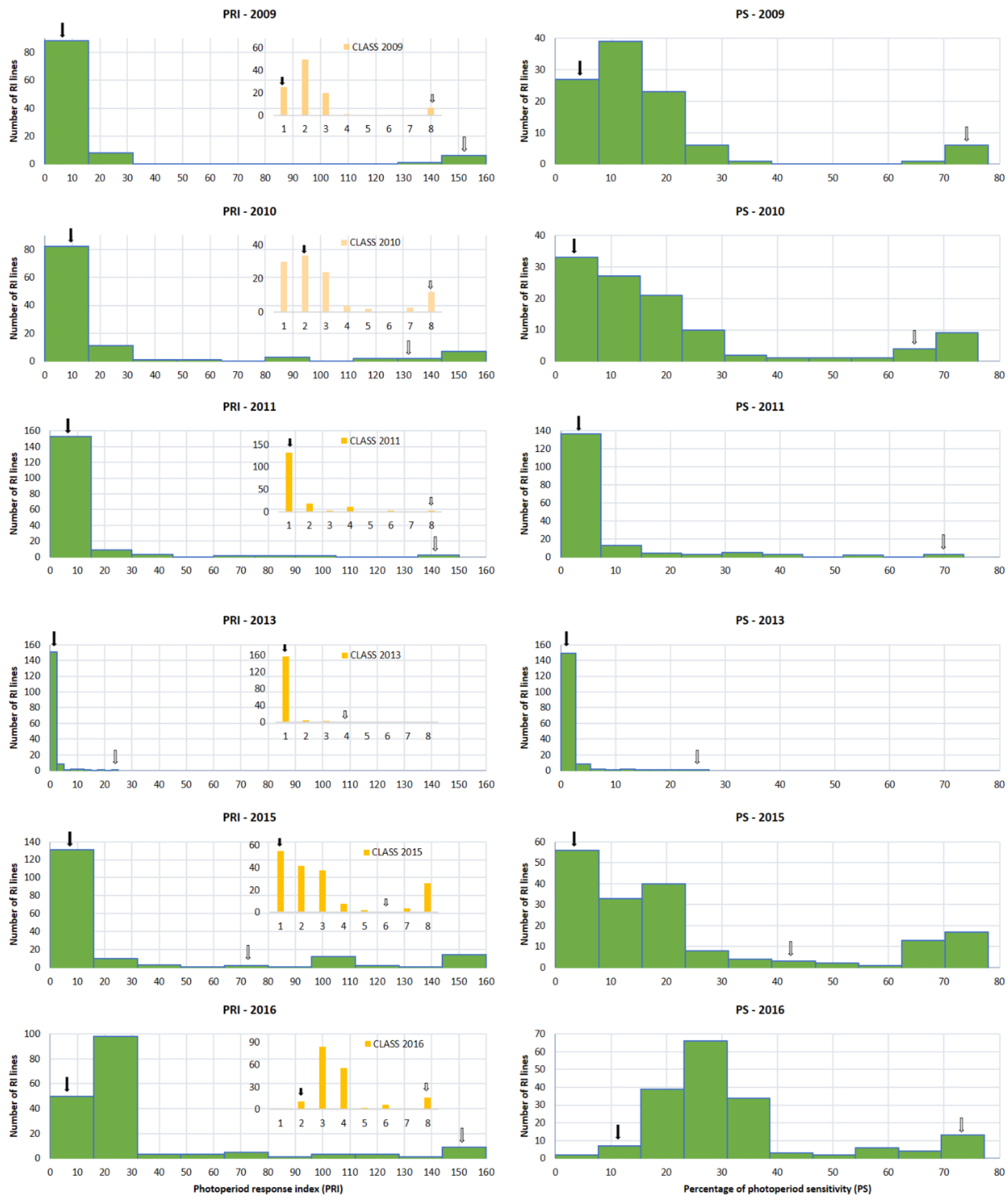


**Fig. S4.** Distribution of the Photoperiod Response Index (PRI), response photoperiod classes 1 to 8 (CLASS), and the photoperiod sensitivity (PS) in the RI population of a cross between the Andean cultivar Bolita (indeterminate type II, photoperiod-insensitive) and the Andean landrace PHA1037 (indeterminate type IV, photoperiod-sensitive); where black and white arrows correspond to Bolita and PHA1037 parents, respectively. Response to photoperiod was measured as a relative change in rate of flowering under long and short day-lengths in several years. PRI values close to zero indicate non-photoperiod-sensitive flowering (stable flowering period), while values close to 30 or even higher indicate high sensitivity to photoperiod (non-flowering). PS values close to or lower than 30% could be classified as photoperiod insensitive, while values over 50% indicate high sensitivity to

photoperiod. Grouping response classes 1 and 2 were classified as day-neutral, 3 and 4 as intermediate, and 5-8 as sensitive.

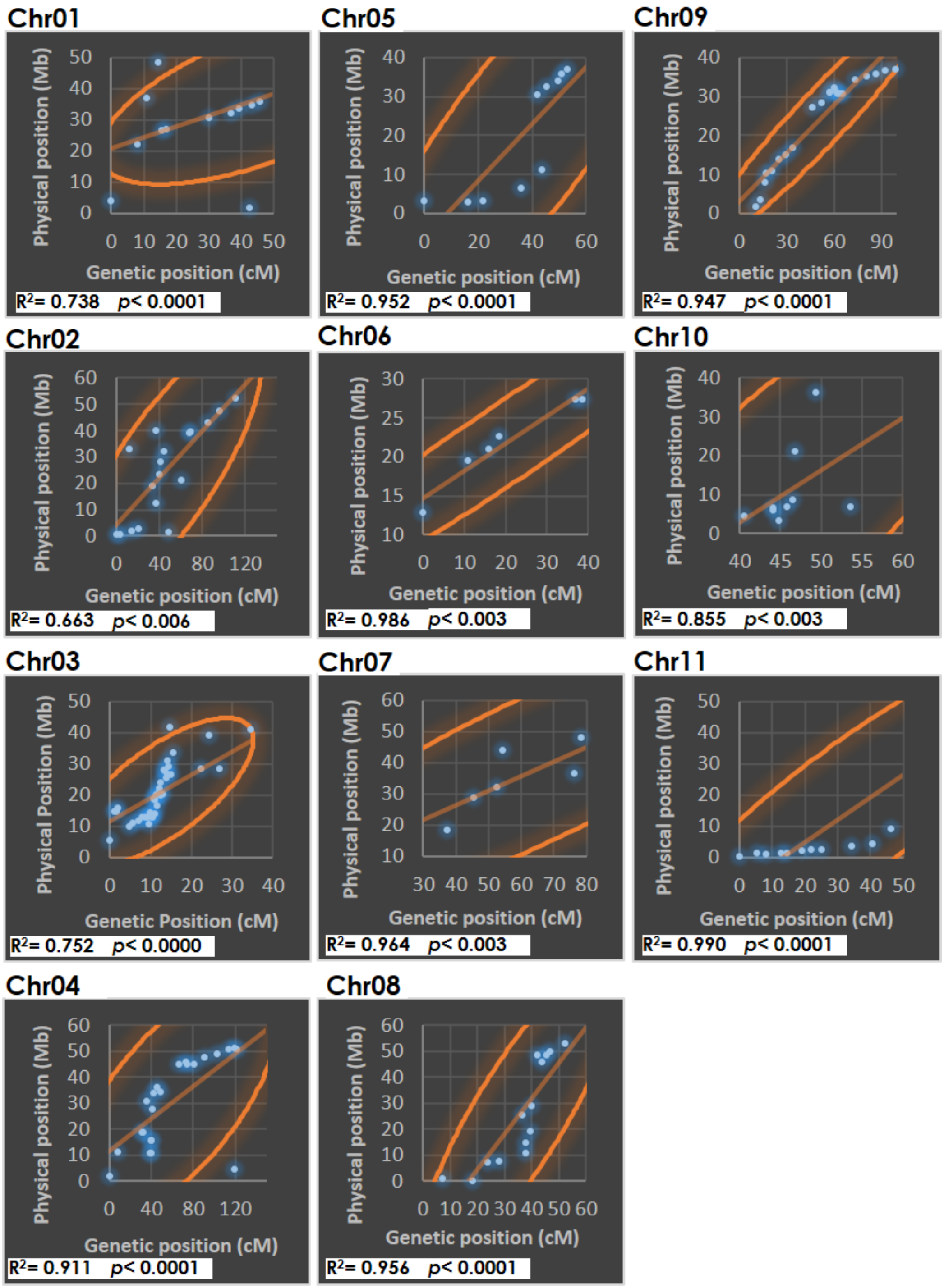


**Fig. S5** Correlation of the genetic and physical positions and its distribution in chromosomal rearrangement regions. The blue dots represent the genetic and physical positions of markers. Total-sample data ellipses are shown as orange, solid curves. The horizontal axis represents the genetic position (cM) of the markers on the genetic linkage map. The vertical axis represents the physical position (Mb) according to the *Phaseolus vulgaris* reference genome (Phytozome). $R^2$ represents the Spearman correlation coefficients between the genetic and physical positions in each chromosome.
